# ConPADE: Genome Assembly Ploidy Estimation from Next-Generation Sequencing Data

**DOI:** 10.1371/journal.pcbi.1004229

**Published:** 2015-04-16

**Authors:** Gabriel R. A. Margarido, David Heckerman

**Affiliations:** 1 Microsoft Research, Los Angeles, California, United States of America; 2 Departamento de Genética, Escola Superior de Agricultura ‘‘Luiz de Queiroz”, Universidade de São Paulo, Piracicaba, Brazil; Ottawa University, CANADA

## Abstract

As a result of improvements in genome assembly algorithms and the ever decreasing costs of high-throughput sequencing technologies, new high quality draft genome sequences are published at a striking pace. With well-established methodologies, larger and more complex genomes are being tackled, including polyploid plant genomes. Given the similarity between multiple copies of a basic genome in polyploid individuals, assembly of such data usually results in collapsed contigs that represent a variable number of homoeologous genomic regions. Unfortunately, such collapse is often not ideal, as keeping contigs separate can lead both to improved assembly and also insights about how haplotypes influence phenotype. Here, we describe a first step in avoiding inappropriate collapse during assembly. In particular, we describe ConPADE (Contig Ploidy and Allele Dosage Estimation), a probabilistic method that estimates the ploidy of any given contig/scaffold based on its allele proportions. In the process, we report findings regarding errors in sequencing. The method can be used for whole genome shotgun (WGS) sequencing data. We also show applicability of the method for variant calling and allele dosage estimation. Results for simulated and real datasets are discussed and provide evidence that ConPADE performs well as long as enough sequencing coverage is available, or the true contig ploidy is low. We show that ConPADE may also be used for related applications, such as the identification of duplicated genes in fragmented assemblies, although refinements are needed.

## Author Summary

Diploid organisms, such as human beings, have two “copies” of each chromosome, whereas polyploid organisms have multiple “copies” (we use quotes to stress that the “copies” are not identical). A key difference between diploid and polyploid organisms is that the “copies” tend to be less similar in polyploid organisms. This difference leads to important differences in the process of *de novo* genome assembly from short fragments of DNA. In particular, when assembling polyploid organisms, contigs corresponding to different copies of the chromosomes can be quite different, and merging them leads to loss of information. Thus, it is important to maintain distinct contigs, even though they correspond to copies of the same chromosomal region. An important step in doing so is to determine how many truly distinct copies of a chromosomal region are found in a single contig. For example, if there are 12 copies of a particular chromosome, the possible number of distinct copies could be anywhere from 1 to 12. We call this task “contig ploidy estimation”, and present a method for accomplishing it. This set of methods is useful for the *de novo* assembly of complex, polyploid genomes such as sugarcane, switchgrass, and wheat.

This is a *PLOS Computational Biology* Methods paper

## Introduction

Complete genome *de novo* sequencing and assembly is a major initial step in understanding the underlying genetic architecture of important traits in any species [1–3]. Reliable reference genomes are pivotal for finding genetic variations such as single nucleotide polymorphisms (SNP) and insertions/deletions (indels), which bolster downstream applications such as genome-wide association studies, population genomics and comparative biology [4–7]. Genetic breeding programs also benefit from reference genomes through the identification of superior promoters and genes [8], which may later be channeled to transformation applications. There have been many algorithmic developments yielding a myriad of software for assembly of the large amounts of short reads generated by next-generation sequencing technologies, mainly developed under a haploid or diploid mindset [9–12]. Such methods have been successfully applied to many diploid species for which high quality or draft reference genomes are now available [13–15]. In the case of more thoroughly studied species, particularly *Homo sapiens*, current work involves resequencing of a large number of individuals to characterize genetic variation, as illustrated by the 1000 Genomes Project [16].

With these already established methodologies, research is moving to larger and more complex genomes [17]. Plants are particularly challenging, due to the highly repetitive nature of their genomes, combined with widespread occurrence of different forms of polyploidy, such as allopolyploidy (*e*.*g*., wheat, many species of the genus *Brassica* and some types of cotton) [18], autopolyploidy (such as potato, sugarcane and switchgrass) [19] and even paleopolyploidy (*e*.*g*., *Arabidopsis* and maize) [20]. To circumvent many of the difficulties arising from such high complexity, researchers have undertaken approaches such as sequencing doubled monoploids to reduce heterozygosity, as was done with autotetraploid potato [21], chromosome sorting and/or bacterial artificial chromosome (BAC) sequencing, such as done for allohexaploid wheat [22]. These approaches are time and resource consuming and may not be applicable to all species. On the other hand, whole genome shotgun (WGS) sequencing is a much less costly option that does not require extensive library preparation or cloning efforts, but in turn results in more fragmented assemblies [23]. Hybrid approaches, for example combining WGS with BAC sequencing, can be used to balance the tradeoffs.

When a genome is assembled with WGS data, regions where two or more copies are similar to each other result in a collapsed assembly, such that a single contig represents more than one haploid segment. Unfortunately, such collapse is often not ideal. Keeping contigs separate can lead to improved assembly due to simplification of downstream analyses such as genome finishing. Maintaining separation will also yield a more detailed view of the polyploid genome, which in turn can lead to (*e*.*g*.) insights about how haplotypes influence phenotype.

Here, we describe a first step in avoiding inappropriate collapse during assembly. Our goal is to identify the number of potentially collapsed haplotypes in any given contig, affording information for subsequent efforts aimed at properly separating distinct genomic segments. In particular, we describe a method to estimate the ploidy of a contig. The algorithm, called ConPADE (Contig Ploidy and Allele Dosage Estimation), estimates ploidy using the relative proportions of alleles in heterozygous positions along with a learned model of measurement error. We evaluate the accuracy of ConPADE with both simulated and real datasets, and show how the approach can also be used for allele dosage estimation in polyploid species. Our approach is applicable to shotgun data from an entire genome or from subsets of a genome, and is valid as long as there is random sampling of all segments potentially collapsed into a single contig—that is, there is no preferential sequencing or higher coverage from one or another genomic segment. It is fundamentally different from copy-number detection algorithms, which are designed to look for departures from a normal situation of diploidy [24,25], and from SNP calling algorithms, which find variants based on the assumption of diploidy [26], or assume an user defined ploidy level [27,28].

At first glance, it may seem that ploidy estimation is trivial when the number of homoeologous copies is known prior to assembly. However, for aneuploid species such as sugarcane, the number of copies varies from one chromosome to another [29]. Furthermore, even if the number of homoeologues is the same for each chromosome, in a given region, some homoeologues will be identical, some will be different, and some will be lacking the region altogether. Consequently, if a region has *k* homoeologues, the contig ploidy in that region can range from 1 to *k*.

ConPADE should also prove useful in estimating the true ploidy of an organism. With lower sequencing costs and better assembly methods, we anticipate that many species for which there is no genomic information will undergo genome sequencing. Even for more well-known species, there may only be rough ploidy information available. In all these situations, it would be beneficial to have information on the ploidy of specific contigs.

## Results

### HiSeq Error Model

**Fig 1:**
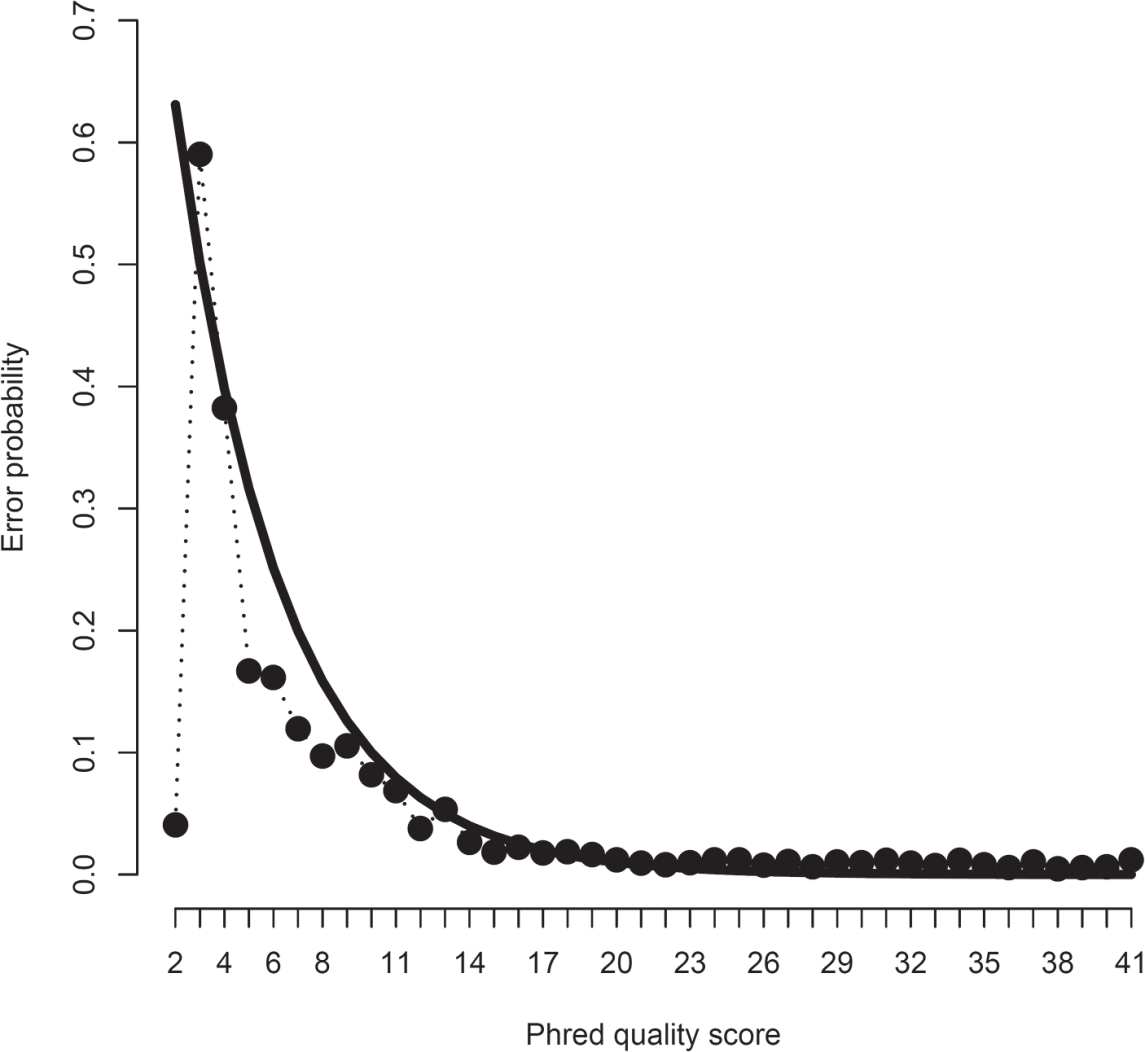
Sequencing error probabilities. Observed sequencing error probability as a function of the Phred quality score (dots connected by the dotted line) and the expected error probability according to the expression 10^(−*QS* /10)^, where *QS* represents the quality score (solid line). There is overall agreement between empirical observations and theoretical expectation, expect for the quality score of 2.

Examination of the quality score distribution showed an apparent excess of bases with quality 2 (S1 Fig), the lowest possible value, indicating that the base calling algorithm could not reliably call a nucleotide for over 12% of the cases. Furthermore, assessment of realized error probabilities showed a strong deviation from the expected value particularly for a quality score of 2 (Fig 1). These observations indicate that many sequenced nucleotides were inappropriately assigned a poor quality score. One approach for dealing with low scores would be to trim reads or remove entire reads. In Section *Simulations*, however, we show that such reads can be incorporated into the analysis of ploidy, provided an appropriate error model is used.

**Fig 2:**
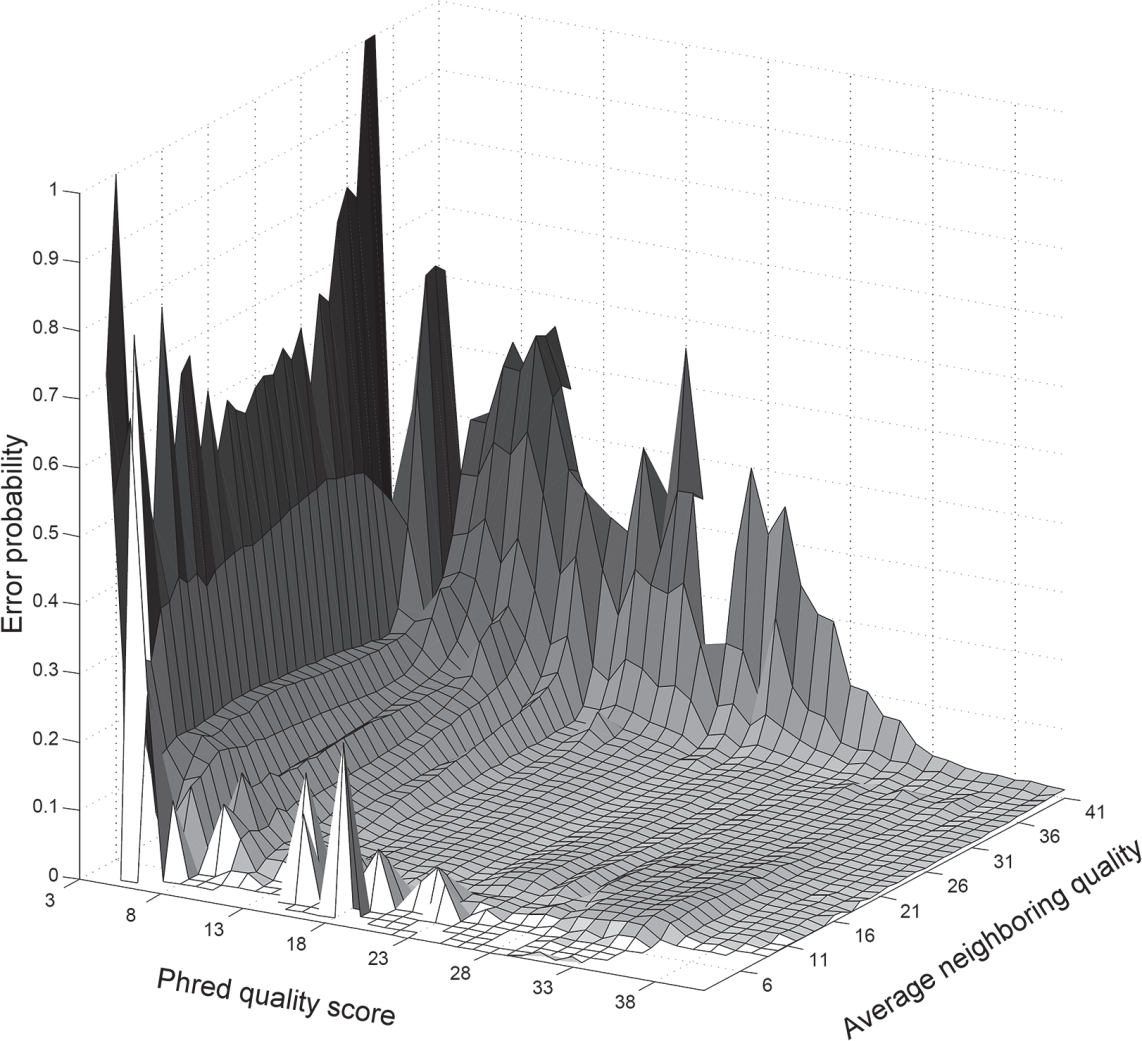
Error probability surface depicting predictive influence of the average neighboring quality score. Note that Phred quality score 2 was not included.

Sequencing quality of the neighboring region gives further indication of whether a given nucleotide can be relied upon. In particular, it is known that nucleotides with high quality scores can nonetheless be of lower actual quality when surrounded by a region of low quality [30]. Our observed error probability surface over the nucleotide quality score and the average neighboring quality score does indeed show a slight bump in the plot for high quality nucleotides in a poor quality region (bottom part of Fig 2). More interestingly, however, we have also observed that the error probability is significantly increased when an intermediate quality nucleotide is surrounded by a high quality neighborhood.

Modeling the sequencing error probability by taking into account quality score and neighboring quality score features, as well as specific nucleotide substitutions and the preceding sequenced nucleotides, resulted in a substantially superior fit over the quality score information alone, as shown by cross validation analyses. By doing so, we were also able to keep all aligned reads in the dataset, without removing allegedly low quality bases. We note that the modeling of sequencing errors is similar to the approach taken by some SNP calling methods, such as the one employed by GATK [27]. Evaluation of the error model on ploidy estimation is given in Section *Simulations*.

### Summary of the Ploidy Estimation Model

**Fig 3:**
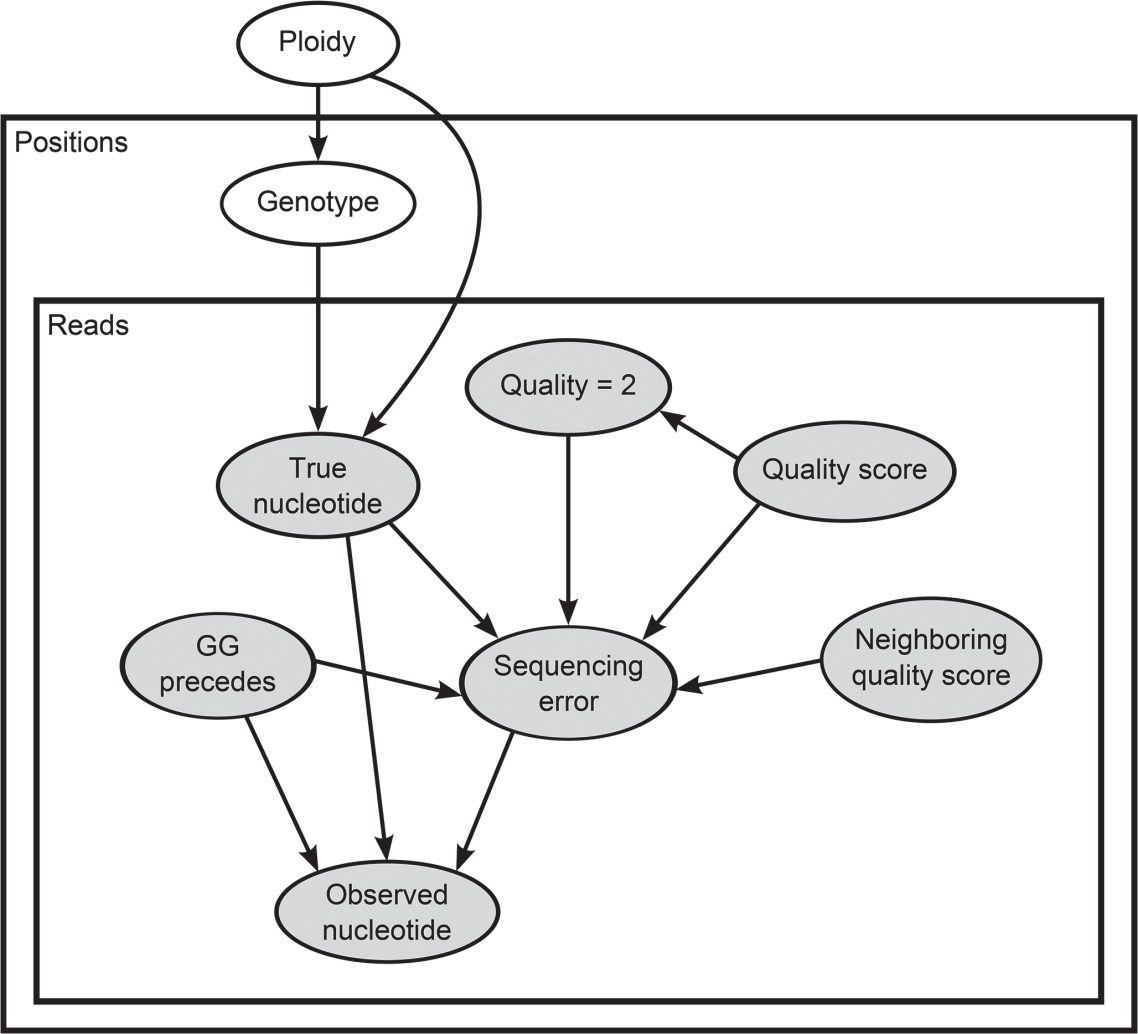
The graphical model for ploidy estimation and variant calls. Each node represents a variable. Edges represent probabilistic dependencies. Each node is associated with a probability distribution of the corresponding variable conditioned on the variables corresponding to its parents. Variables within the same plate (rectangle) are replicated according to the number of positions in a contig (the “Positions” rectangle) or the number of reads overlapping a given position of a given contig (the “Reads” rectangle). Shaded variables represent the HiSeq error model, which is a component of the ploidy estimation model.

In our model, we assume that there are at most two possible alleles at any given position. For a genomic region with any given level of ploidy, herein denoted *M*, heterozygous sites in the genome can hold varying proportions of these two alleles. As an example, all heterozygous positions in a diploid region will display the two alleles in a 1:1 ratio. Alleles in a triploid region can be present in 2:1 or 1:2 ratios. A tetraploid can display the ratios 3:1, 2:2 and 1:3. In general, the number of heterozygous possibilities is *M*−1. This structure is captured in the generative model displayed in Fig 3. Because particular contigs or scaffolds can represent a varying number of copies in a polyploid individual, due to collapsing during assembly, this model assumes ploidy is constant along each contig, instead of along the entire genome.

For a given contig or scaffold, the genotype at each position refers to the ratio between the two alleles. First, we define the probability of there being a SNP in any position as *P*(*SNP*). We then assume a uniform distribution for all possible heterozygous proportions, as done by others in a polyploid genotyping context [31], which corresponds to setting $P\left(G=g\right)=\frac{P(SNP)}{M-1}$, where *G* denotes the dosage of the first allele, with *g* = 1, ⋯, *M*−1. The dosage of the other allele is consequently *M*−*g*. The order of alleles is defined arbitrarily without loss of generality. For *g* = 0 and *g* = *M*, which correspond to a position with no true variation, we set $P\left(G=g\right)=\frac{1-P(SNP)}{2}$, such that both possibilities are uniformly distributed. Lastly, for each read at each position, we assume that the true (unobserved) nucleotide follows a Bernoulli distribution with probability equal to the proportion of the first allele—that is, $T=\text{Bern}\left(\frac{g}{M}\right)$, where *T* takes on the value 1 or 2 representing the first or second allele. Our previously learned HiSeq error model is then plugged into this ploidy model.

Our model takes into account information from all genomic positions of a given contig/scaffold, having a nested model for all reads covering each position. We use the model to infer the probability of observing each particular nucleotide in the dataset for all possible genotypes for any given ploidy, and subsequently infer the ploidy that maximizes the likelihood of the observed data. The default implementation sets a uniform prior for every ploidy, but prior information can be easily incorporated.

After selecting the ploidy with highest posterior probability, an estimate of the most likely genotype can be obtained for each individual position. More or less conservative thresholds, as well as other optional filters, can be used to call variants—that is, heterozygous genotypes.

The model is described in more detail in *Methods*.

### Simulations

To evaluate the performance of this ploidy estimation model, we simulated data from several different scenarios and applied the ConPADE method to each of them. We evaluated contigs with ploidy ranging from one to 16, consistent with the vast majority of real data. For example, potato is tetraploid (*Solanum tuberosum*, 2n = 4x = 48) [21], sweet potato is hexaploid (*Ipomoea batatas*, 2n = 6x = 90) [32], and sugarcane cultivars present different levels of ploidy, from 5X to 12X or more, with further aneuploidy (*Saccharum spp*, 2n = ca 110 to 120) [33]. We simulated coverage levels varying from 10X per copy, which is typically less than optimal, to a coverage of 75X per haploid copy, which is higher than the usually employed datasets, although currently practicable given the continuously decreasing costs of next-generation sequencing data. Some eukaryote genome sequencing projects have already used such depth of coverage [34,35].

To assess the effect of different levels of sequencing coverage, for each ploidy, we initially simulated a 10 Mb long contig, which is a long enough sequence to contain thousands of SNPs spaced at a reasonable distance. Our goal was to isolate the effect of sequencing coverage from contig length in this first set of simulations. Results from such simulations are shown in S2 Fig and S1 Table. When using the full error model, ploidy was correctly estimated in each experimental condition for depths of coverage of 15X and above. For the lowest coverage of 10X per haploid segment, ploidies from one to 11 were correctly called. However, for generated ploidies of 12 through 16, estimates were consistently downward biased such that the estimated ploidy was one unit below the underlying truth. Indeed, with low coverage, higher ploidies are expected to be harder to distinguish from one another, because of the increasingly smaller distances between the dosage-to-ploidy ratios. When using a “naïve” error model that only take quality scores into account, ploidy estimate errors were more substantial. For 10X coverage, the contig with ploidy eight was estimated as having ploidy 16, indicating that many sequencing errors were not correctly weighted by the error model. Furthermore, there was an error for coverage of 15X, because ploidy 15 was called as 14, which did not happen with the full model.

Having called the most likely ploidy for each simulated scenario, we then checked whether the dosages of both alleles were correctly inferred (S2 Fig and S1 Table). When using the full model, correct dosage was obtained for over 95% of the SNPs for levels of coverage of 50X and 75X, across every simulated ploidy. As expected, dosage calling accuracy decreased with decreasing coverage, reaching a minimum of 76.12% for ploidy 16 at 15X coverage. With the exception of two cases (ploidy eight, with coverage levels of 50X and 75X), percentage of correct dosages was always equal or lower when using the naïve error model. Discrepancies in dosage calling accuracy between the two models tended to increase with higher ploidy and lower coverage, such that in the most extreme situation of ploidy 16 at 15X coverage, the full model made 3.24% more correct calls. Higher levels of coverage resulted in more similar accuracies, with differences mostly below 2%. False positive and false negative levels of variant calling were extremely low for all simulations, never going above 1.7%.

**Fig 4:**
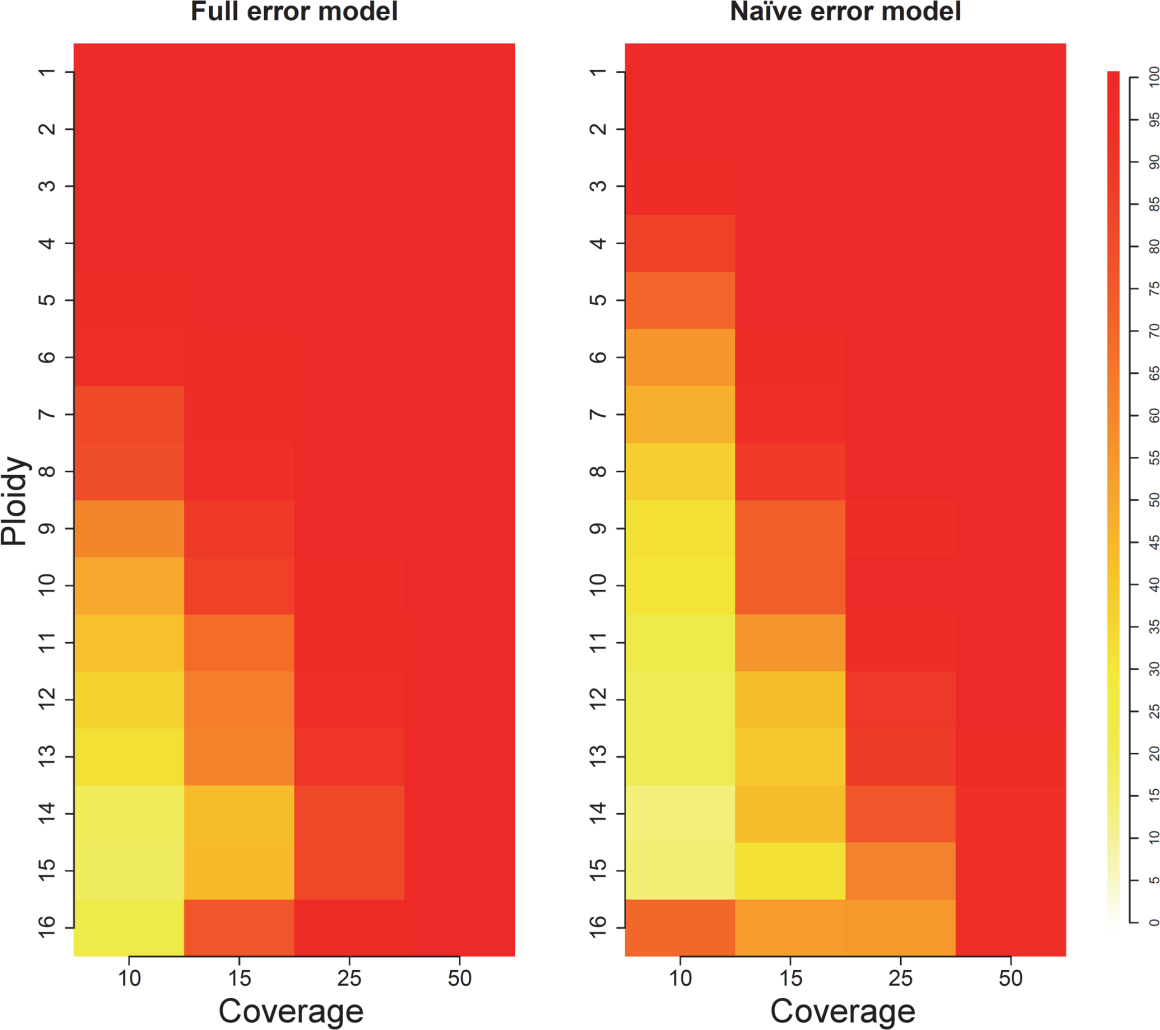
Coverage simulation results. Color in each cell indicates the percentage of correct ploidy calls, out of 100 simulations of 200 kb-long contigs for each ploidy level.

These initial results indicate that ploidy estimation is more challenging for higher levels of ploidy, and that there can be random variation leading to errors in estimates. In order to provide estimates of ploidy calling accuracy with varying coverage levels, we simulated 100 sets of 200 kb-long contigs for each ploidy level and evaluated the performance of ConPADE in each situation. Results are shown in Fig 4 and S2 Table, where warmer colors indicate higher ploidy estimation accuracy. For a coverage level of 50X, ploidy estimation with the full error model yielded correct results for all 100 simulated contigs, for all ploidy levels. The accuracy of ploidy estimation decreased with decreasing coverage, particularly for higher ploidies. In that context, we note that the lowest accuracies were 83%, 44% and 19% for coverages of 25X, 15X and 10X, respectively, for a ploidy of 15. It is interesting to note that, whenever ploidy was incorrectly called, the estimated and actual ploidy usually differed by at most two, and never more than three. Specific ploidy calls for coverage of 15X are found in S3 Fig and S3 Table.

With regards to allele dosage estimation, we observed that 50X coverage resulted in correctly estimated dosages for 94% or more of the SNPs, for every simulated ploidy. Again, accuracy was reduced with decreasing coverage, reaching values as low as 86.08%, 75.56% and 64.32% for coverages of 25X, 15X and 10X, respectively, for the ploidy of 16. Finally, the sensitivity of variant detection was high for all simulated situations, with false negative rates for SNP calling ranging from zero to 7.05%. As expected, higher false negative rates occurred for the lower coverage levels. Similarly to what we observed for incorrect ploidy calls, estimated and actual allele dosage calls differed by one or two. Particular dosage calls for a ploidy of 15 with sequencing coverage of 15X are shown in S4 Fig and S4 Table. The latter table also shows that dosage calling accuracy was lower for intermediate allele ratios, because of the larger variance of the Bernoulli distribution for intermediate probability values. For example, a SNP with allele ratio 7:8 resulted in lower dosage accuracy than a SNP with allele ratio 13:2. It is worth mentioning that we conducted some of the above simulations with 10 Mb-long contigs, but saw little improvement over the 200 kb length. Similarly, coverage of 75X was only slightly superior to 50X coverage.

When using the naïve error model, ConPADE achieved substantially lower ploidy estimation accuracy, especially for coverages of 10X and 15X at intermediate ploidy levels (Fig 4 and S2 Table). Even for 25X coverage, with ploidies 14 and above, the naïve model failed to provide correct estimates in many instances. Investigation of the likelihoods showed that the naïve model did not appropriately control for the influence of sequencing errors, which led to overestimated ploidy levels. This can be seen more clearly for the ploidy of 16 at 10X coverage, which displayed inflated accuracy due to the fact that this error model up-biased ploidy estimates. In the cases where ploidy was correctly inferred, the naïve and full error models showed only minor differences in dosage calling accuracy. For ploidies above three, the full model always produced more correct dosage calls than the naïve model, with differences ranging from 0.10% (ploidy level four, coverage of 50X) to 2.83% (ploidy 16, 15X coverage). The sensitivity of variant calling of the naïve error model was greater than that of the full error model, particularly for low coverage situations, which is indicative of its less conservative nature (S2 Table). With higher coverage levels, both models had similar false negative rates of SNP discovery.

**Fig 5:**
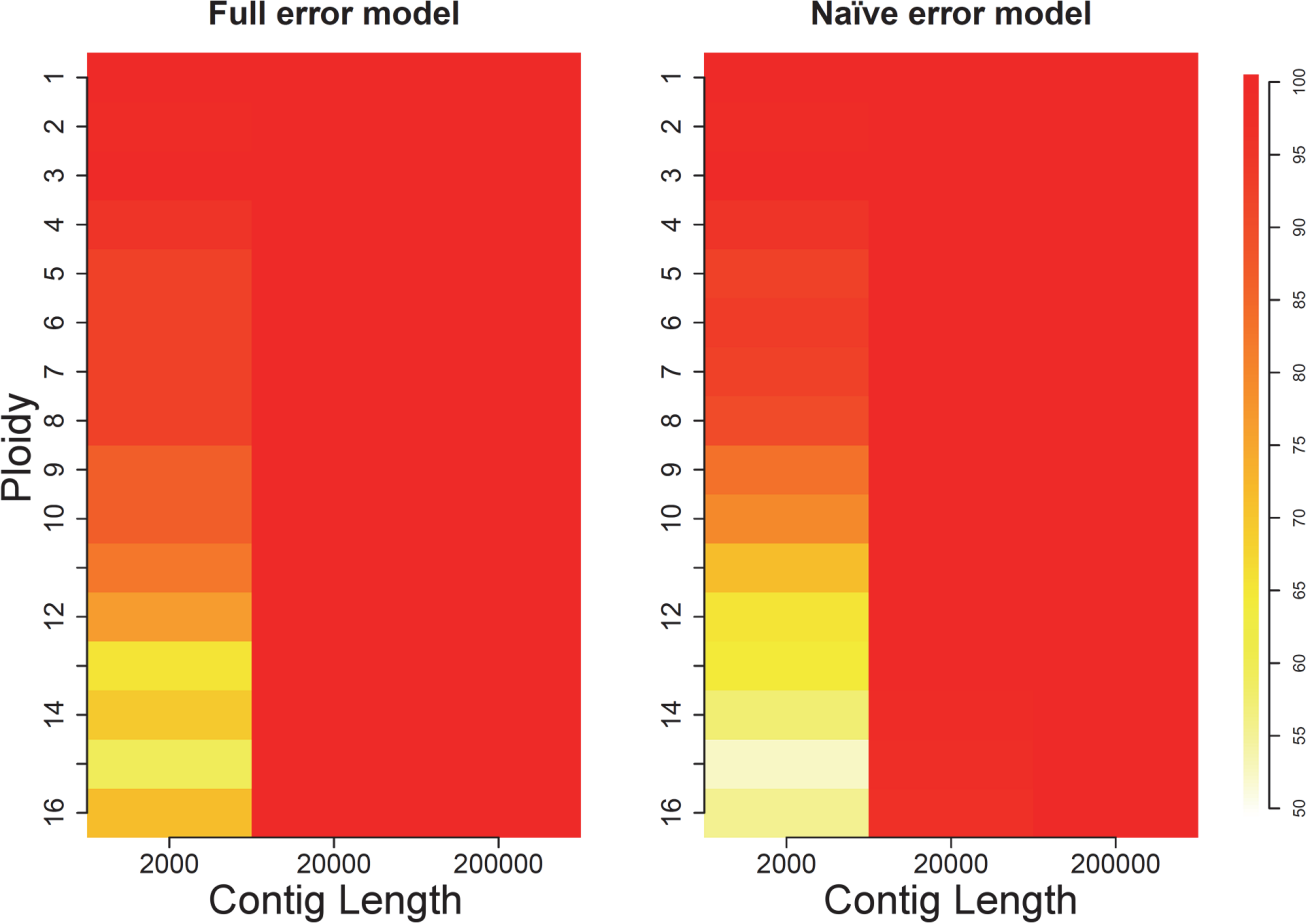
Length simulation results. Color in each cell indicates the percentage of correct ploidy calls, out of 100 simulations of contigs sequenced at 50X coverage for each ploidy level.

Because a coverage level of 50X resulted in correct estimated ploidies and high dosage estimation accuracy in the previous simulation sets, while still being viable in practice for *de novo* genome assembly efforts, we chose this value for more detailed simulations regarding contig lengths, the results of which are shown in Fig 5 and S5 Table. For contigs of 20,000 nucleotides or longer, which in this case contain 100 informative variants on average, the full model resulted in correct ploidy estimates in every simulated dataset. For very small contigs, containing only a handful of SNPs, ploidy estimation accuracy decreased with increasing ploidy, with 60 to 70% of correct estimates for ploidies over 13. Once more, the percentage of correctly called dosages was above or close to 95%, providing evidence that, given the correct ploidy, dosage estimation with this level of coverage is accurate. False negative rates were higher for shorter contigs, due to the fact that there was lower or no read coverage on the edges of contigs (S5 Table).

ConPADE yielded less accurate ploidy estimates for short contigs when combined with the naïve error model, especially for higher ploidy levels, with differences of up to 16% between the two models, as was the case for ploidy 16. Contigs of 20,000 bases or more displayed similar results between both error models, with slightly lower dosage estimation accuracy for the naïve model. Again, false negative rates tended to be slightly lower with the naïve model, indicating a more conservative nature of the full model, because the simpler model resulted in a larger number of SNP calls (S5 Table).

Finally, to assess the ploidy estimation accuracy profile on different coverage levels and contig length combinations, we downsampled the latter simulations to achieve coverages of 25X and 15X, for ploidies varying from one to eight, which are the most commonly observed in practice. The results are shown in S5 Fig. There was little effect of contig length for 50X sequencing coverage, and ploidies were called with a minimum accuracy of 93% across all conditions. When coverage dropped to 25X, contigs of 2,000 bases yielded correct estimates 75% or more of the time for ploidies of seven or less, and 20,000 bp contigs performed almost as well as the longest ones for all ploidy levels. Lastly, coverage of 15X allowed accurate estimation only for ploidies lower than or equal to four, in the case of short 2,000 bp contigs, while the intermediate contig length still afforded reliable ploidy estimates in all scenarios.

Collectively, these simulation results show that high ploidy levels can be reliably estimated only with high sequencing coverage, even for long contigs. Short contigs require higher depths of coverage, but still produce useful results in low coverage circumstances when the true ploidy is low.

### Switchgrass Dataset

To further evaluate our ploidy estimation model, we analyzed a real dataset for Switchgrass. Switchgrass (*Panicum virgatum* L.) is a member of the grasses (*Poaceae* family) and has recently gained importance as a source of bioenergy [36]. It is believed that most grasses are polyploids, with occurring instances of autopolyploidy, allopolyploidy and aneuploidy [37]. Switchgrass, in particular, is comprised of (pseudo)tetraploid and octoploid genotypes, some of which are commonly aneuploid and display genome instability [38].

We used the preliminary *Panicum virgatum* AP13 genome reference as a test case for our model. AP13 is a tetraploid clone with two sub-genomes, which are highly similar in some genomic segments, due to the recentness of the polyploidization event [36]. The reference genome consists of an assembly of 15X coverage of the expected 1.4 Gb genome with Roche 454 data, which resulted in a total of 410,030 contigs with L50 of 4.2 kb. The contig length distribution from this assembly is shown in S6 Fig. Total assembly length was 1.358 Gb. Next, we downloaded from the NCBI Sequence Read Archive whole genome shotgun reads from the same genotype, obtained through the Illumina HiSeq 2000 platform, in a total of 106.4 Gb of sequence data, and aligned all read pairs against the reference genome.

From 5,000 randomly sampled contigs, 4,879 had at least one aligned read pair and could be analyzed. Average sequencing coverage was 232.2X. The distribution of estimated ploidies for these 4,879 contigs showed that almost 90% of them (4,381) represented more than four collapsed haploid copies. In particular, we observed a peak at ploidy eight. The fact that many contigs displayed ploidies higher than the expected organismal ploidy likely indicates contigs containing paralogous regions collapsed into a single reference sequence.

Overall, we called 134,464 variants within the contigs, with an average density of one SNP every 47 nucleotides. Manual examination of the called SNPs showed that, without explicitly enforcing any filter or threshold, all heterozygous positions had at least three reads supporting the minor allele, from a total of at least seven overlapping reads. We also performed variant calling with GATK [27] and obtained a density of one SNP every 60 bases. We note that these SNP densities may be inflated due to homoeologue collapse and may not reflect exclusively allelic variation.

**Fig 6:**
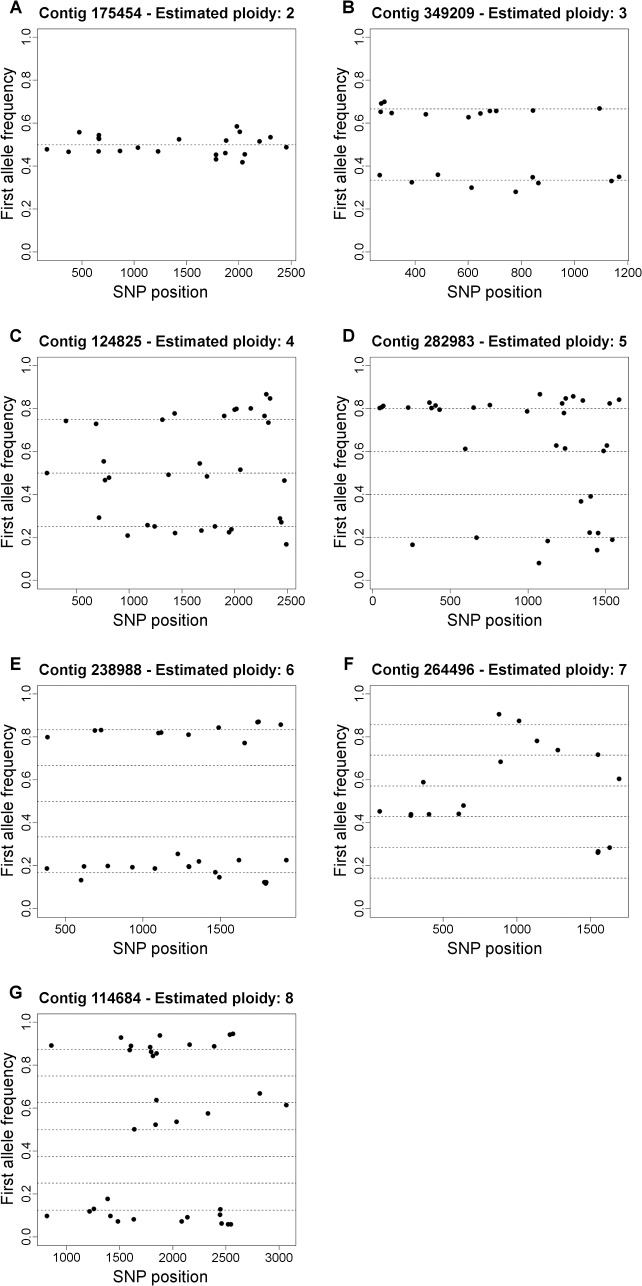
Observed allele ratios of variants called by ConPADE for switchgrass contigs with various estimated ploidies. Each dot represents a significantly identified variant position. For each estimated ploidy, dashed lines represent expected genotypes.

Fig 6 displays observed allele ratios for called variants, from examples of contigs representative of each estimated ploidy. Allele ratios were in agreement with possible values given estimated ploidies, as visualized by distances of individual SNPs from the dashed lines. It is also interesting to note that allele ratios provide a rough guide to sequence diversity within a given segment. For instance, contig 238988 had an estimated ploidy of six and virtually all called variants displayed an allele ratio of 1:5 (Fig 6E). In other words, most identified SNPs presented only one copy of the less frequent allele. A closer look at the reads aligned against a region containing some of the variants in that contig provides a picture of how the alleles are organized in haplotypes (S7 Fig). Interestingly, in this case, most minor alleles are linked to each other in the same reads, forming a single haplotype. This haplotype is present in a roughly 1:5 ratio with regards to the underlying reference sequence.

**Fig 7:**
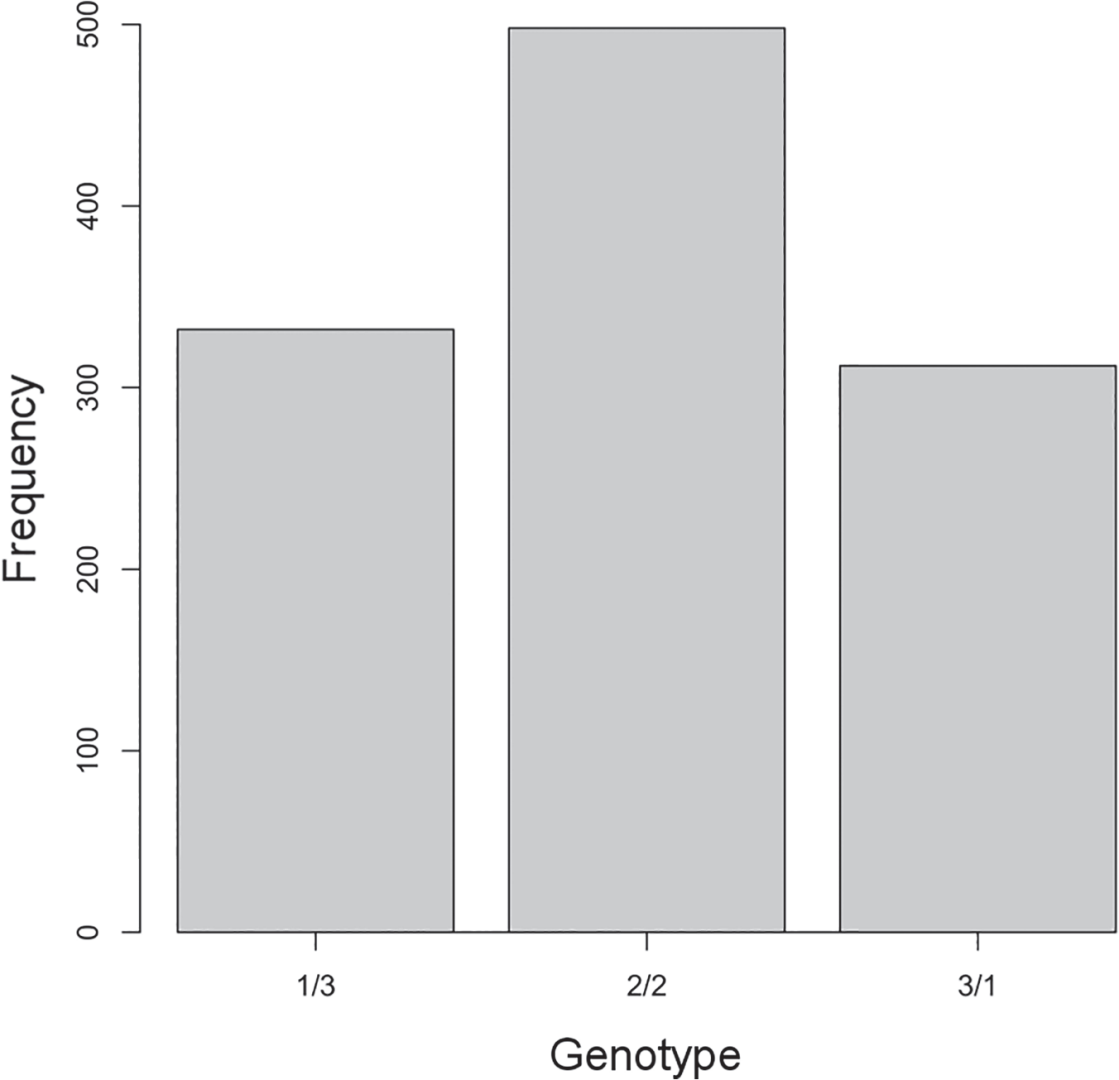
Genotype distribution for switchgrass contigs with a ploidy estimate of four. Bars represent the frequency of each SNP genotype, for all identified variants in contigs estimated to have ploidy four.

It is also interesting to investigate the distribution of estimated genotypes, because doing so can provide insights about how the genome is structured. Because genotype AP13 is expected to be a tetraploid, we focus on contigs with an estimated ploidy of four (Fig 7). The apparent excess of SNPs with genotype 2/2 possibly reflects the (pseudo)tetraploid nature of this particular switchgrass individual, such that these SNPs likely arise from differences between the two sub-genomes. Additionally, this result provides empirical evidence that our uniform parameterization for genotypes does not excessively constrain *a posteriori* estimates of allele dosage, given moderate sequencing coverage levels.

### Wheat Dataset

In the Switchgrass analysis, we saw examples where the estimated ploidy of a contig was greater than the known organismal ploidy due to potential collapse of non-allelic regions in the assembly. We also investigated possible collapse in wheat. Common wheat (*Triticum aestivum* L.) is an important food source, cultivated worldwide to provide carbohydrates and protein for human consumption. The genome is allohexaploid (2*n* = 6*x* = 42) containing three related subgenomes, denoted A, B and D. It is believed that the A genome was donated by a species related to *T*. *urartu* (2*n* = 2*x* = 14), the B genome from a relative of *Aegilops speltoides* (2*n* = 2*x* = 14), and the D genome from *Aegilops tauschii* (2*n* = 2*x* = 14). Cultivated common wheat thus has genomic constitution AABBDD [39]. The complete polyploid genome is 17 Gb in length [34].

Because of its size and complexity, a draft sequence of the wheat genome was created by sequencing and assembly of isolated chromosome arms, instead of a complete *de novo* genome assembly. Chromosome arms were sequenced with Illumina short read technologies and assembled with ABySS [40]. Owing to the employed strategy, based on physical separation of individual chromosome arms, the true ploidy of each partial assembly is one.

To investigate the effectiveness of ConPADE in that situation, as a validation procedure, we initially applied it to sequence data from the large arm of chromosome 5D—that is, chromosome 5 from the subgenome D. This data contains 236.8 Mb of sequence, with a contig L50 of 2,647 bp, and is expected to cover roughly half of the complete long arm of chromosome 5D.

**Fig 8:**
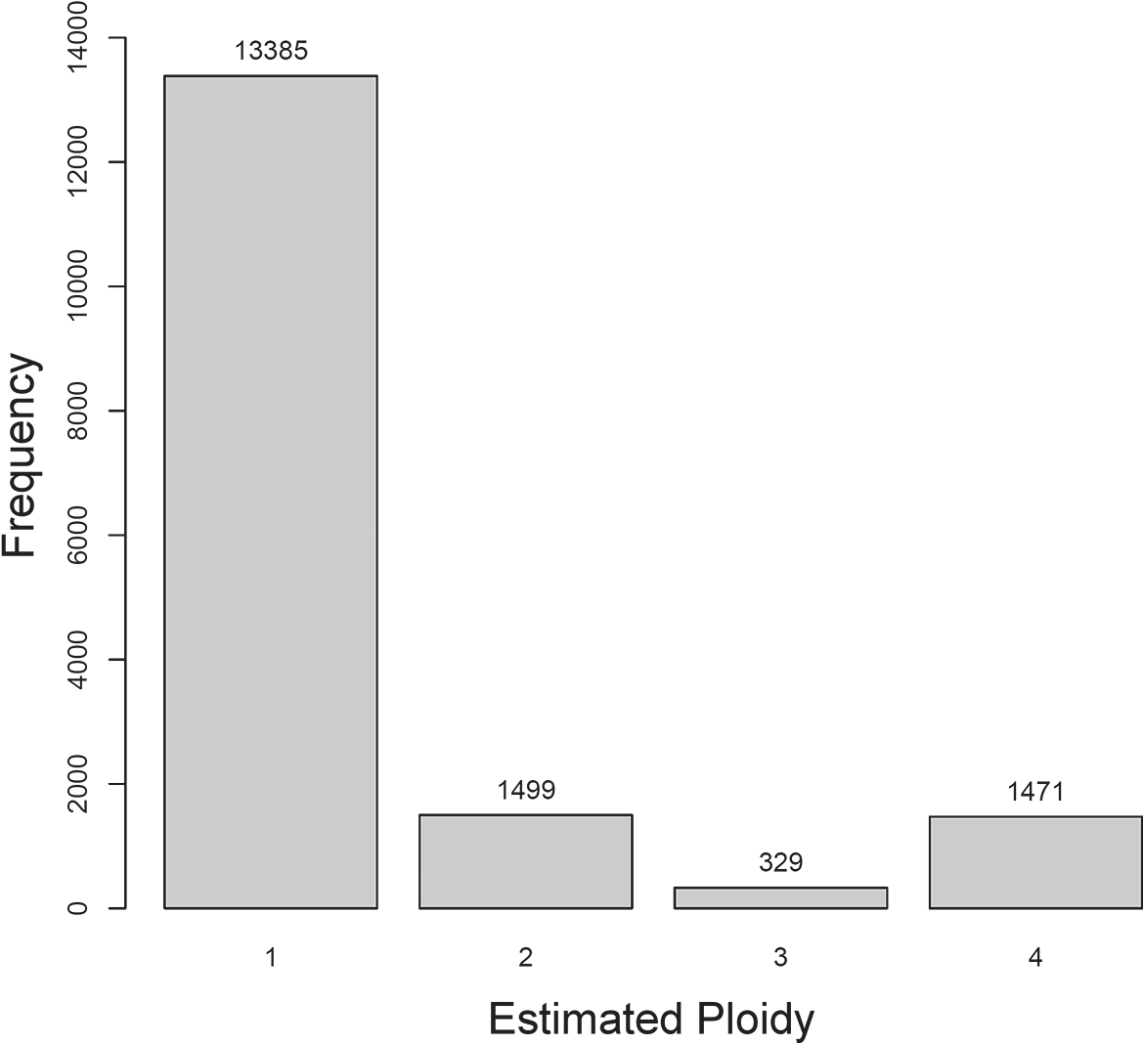
Ploidy estimate distribution for common wheat chromosome arm 5D contigs. Bars represent the frequency of each ploidy estimated by ConPADE, for a set of 16,684 wheat contigs from the *de novo* assembly of chromosome arm 5D.

After stringent read alignment, we could evaluate the ploidy of a set of 16,684 contigs with varying levels of coverage. More than 80% of the contigs (13,385) were confirmed to have a ploidy of one, that is, were inferred to represent a single haploid segment. Contigs with a ploidy level of two represented almost 9% of the total (1,499), as did contigs with a ploidy of four (1,471). Only 329 contigs (1.97% of the total) had an estimated ploidy of three (Fig 8).

Investigation of 30 contigs with estimated ploidy above one revealed that most contain repetitive DNA sequences, with enrichment for known mobile elements, particularly transposons and retrotransposons, ribosomal RNA genes, centromeric and telomeric sequences (S6 Table). Furthermore, common wheat has been shown to exhibit intrachromosomal gene duplication at higher degrees than other grasses, likely arising by tandem duplication. Even more importantly, there is evidence that the current individual chromosome arm assemblies may underestimate the occurrence of gene duplication, due to inappropriate collapse of very similar duplicates [40]. Our ploidy estimation results provide candidates of this phenomenon for further investigation.

To gauge the applicability of ConPADE to an allopolyploid of known genomic origins, we fashioned the scenario that would be obtained had the wheat genome been sequenced via a whole genome shotgun strategy. With this goal, we pooled sequencing reads from the large arm of chromosomes 5A, 5B and 5D and assembled them following a strategy similar to that employed in the published assembly of the wheat genome [40]. This assembly yielded 771.3 Mb of sequence, with a contig L50 of 2,253 bp.

As a benchmark for our method, we aligned these assembled contigs to the three separate published assemblies with BLAST [41], assessing in how many of the subgenomes any newly assembled contig was present. Interestingly, roughly 99% of the analyzed contigs (43,461 out of 43,819) were found in a single subgenome of the three original assemblies, providing evidence that the three wheat subgenomes are different enough that the assembler was able to separate them into different contigs.

Application of our method to this dataset revealed that approximately 93% of the contigs had a ploidy estimate consistent with what was expected based on subgenome assignment by the previous benchmark alignments (Table 1). In the case of contigs that could be assigned to a single genome, ConPADE had an accuracy of 93.51%, and most of cases in which the ploidy was incorrectly called were due to subregions of a given contig being contaminated with reads from the other subgenomes, leading to an upward bias in ploidy estimates. In cases where there was collapse of two or the three genomes, accuracy dropped to 68.20% and 58.67%, respectively. However, manual inspection showed that these contigs were usually shorter than 2 kb and had lower coverage. Confining the analysis to contigs for which the average coverage was 25X or above yielded 100% correct ploidy calls.

**Table 1:** Ploidy estimation for an artificially combined wheat dataset.

Number of subgenomes in which a contig was found^a^	Estimated ploidy			
	One	Two	Three	Four
One	40,642	244	230	2,345
Two	26	193	44	20
Three	1	18	44	12

Reads from the large arms of chromosomes 5A, 5B and 5D were pooled, assembled and used for ploidy estimation. Only contigs with average coverage of 10X or above, and for which the individual ploidy in a given subgenome was estimated to be one were considered.

^a^Based on BLAST alignments to the individual assemblies.

## Discussion

We have presented a ploidy estimation model and verified with both simulated and real data that it gave correct results when sufficient read coverage was available. We have also observed that it can be successfully applied to variant calling in newly assembled genomes.

When learning the parameters of the HiSeq error model, bacterial data was utilized and any variations from the reference genome for the same strain were regarded as sequencing errors. Even monomorphic bacterial strains may hold some genetic variability, and hence these variations may have erroneously inflated error rates. Moreover, some of the reference genomes were originally assembled from HiSeq data, such that systematic errors may have been considered as ground truth. Nonetheless, given the rarity of such events and the large amount of data used, these sources of inaccuracies should have little effect on the final parameters learned, and as we observed empirically, led to good performance of our model.

An underlying assumption of ConPADE is that ploidy is constant along each contig or scaffold, a good assumption in practice for the task at hand. This is fundamentally different from copy number variant detection algorithms, which look for changes in allele ratios (*e*.*g*., using a Hidden Markov Model) [24] or read coverage [25] to find discrepancies from a reference sequence. The assumption of constant ploidy for any given contig makes inference computationally more efficient than (*e*.*g*.) a Hidden Markov Model that allows for changes in copy number, and is also particularly well suited given shorter contig lengths for polyploid genome assemblies. Furthermore, with the assumption of constant ploidy, all positions (SNP or not) can be included in the model without compromising feasibility.

Ploidy estimation is also commonly done in cancer research. However, because cancer is an abnormality derived from a naturally occurring diploid state, the nature of the polyploidy is vastly different, and methods for ploidy estimation in cancer rely on different assumptions. In the cancer framework, a single haplotype is usually expected to be present in multiple copies. As a consequence, heterozygous positions in long stretches of the genome are expected to display the same allele ratio. Consequently, methods for ploidy estimation in cancer can make use of segmentation algorithms to look for particular regions of the genome departing from diploidy [42,43]. In contrast, for polyploid plants, there is no such restriction. The ancestral genotypes leading to the polyploid genotype under analysis, which are potentially polyploid themselves, may have varying allele dosages. In addition, evolution and/or artificial selection during breeding will drastically alter the genomic constitution and shape different genomic configurations, and ploidy estimation in a plant research context needs to be more flexible to accommodate multiple sources of polyploidy.

Another assumption that deserves attention is the fact that only biallelic variations are considered in the model. Combinatorial possibilities of up to four different alleles in different dosages would result in an exceedingly large number of model states to be fit, which would make analysis infeasible. Nonetheless, there is some evidence that SNPs, even for species with high degrees of ploidy, are generally biallelic [31]. In our default implementation, we assumed a uniform distribution for possible dosages within any given ploidy. Because genotypes being sequenced for *de novo* assembly of important crop species are usually chosen from a pool of bred cultivars, the recurrent cycles of crossing and selection make expectations about allele dosage distribution non-trivial. In other situations, such as when a wild genotype is sequenced, it may be more appropriate to assume an exponential distribution for dosages, due to the presence of rare mutations. When more sequencing and SNP data becomes available for higher ploidy species, the empirical distribution of dosages might become more apparent for many distinct scenarios. As such data is gathered, it is straightforward to change the underlying assumption and incorporate such knowledge into our framework. Accurate information about the dosage of each variant will be very useful for downstream analyses such as association studies, where the number of copies of a given locus can have an effect on a phenotype of interest.

In principle, it is possible to leverage information both from SNPs and indels for ploidy estimation. However, ConPADE utilizes only SNPs, because (1) current sequencing technologies lack sensitivity to identify indels, (2) indel processing is more complex than SNP processing [44] and (3) indels are usually less abundant than SNPs [45,46] and would thus contribute little information for ploidy estimation.

From a computational standpoint, exact inference in our model can be performed efficiently for scaffolds that are millions of base pairs long and covered at high sequencing depth. Probabilities arising from the error model can be cached, enhancing efficiency. Also, because each contig/scaffold is analyzed independently from all the others, execution is easily parallelized.

The single input required by the method is a BAM file with alignments of reads or read pairs against the final assembly. The user has control over which short read aligner to use and how to filter alignments. During our experiments with real and simulated data, only reads that aligned to a unique point in the genome with high mapping quality were included in the analysis to reduce occurrence of spurious alignments. In addition, whenever mate information is available, only pairs aligned with the expected range of distances should be considered.

Because ConPADE is based on allele ratios in heterozygous positions, alignment parameters may have a significant impact on the results obtained. In particular, if two distinct genomic regions are sufficiently different such that the assembly software is able to separate them into two distinct contigs, some of the sequencing reads will ambiguously align with both contigs, and will thus have low mapping quality. This situation can be avoided by excluding low quality reads from the analysis.

It is important to stress that our model accommodates all possible *M*−1 heterozygous allele ratios for a genomic region with ploidy *M*, but it is not necessary that they all be present simultaneously. Furthermore, it is interesting to note that SNPs in which the major/minor alleles are present in a *M*−1 to 1 ratio are the most informative ones, since they cannot be present in lower ploidy levels. Because this particular configuration is expected to be more frequent than others in some cases, we note that this situation affords the ideal ploidy estimation scenario.

Simulation results also indicated that ConPADE works well for contigs of small size, on the order of a few thousand nucleotides in length. This is crucial for de novo assemblies of polyploid genomes, which are naturally more fragmented due to genomic complexity. Although high sequencing coverage is necessary for accurate ploidy and allele dosage estimation, we expect that high coverage data will be available for many species with complex genomes in the near future, affording more reliable results and the chance for important insights into their genomic organization.

Variant calling performance was also good, showing that the error model was slightly more conservative than a simple model that only takes quality scores into account. However, the full model was able to leverage information that would otherwise be disregarded. Such a conservative model that performs well on high coverage situations is naturally suited to a newly assembled genome, where large numbers of reads are usually available due to the difficulty of *de novo* assembly. Notwithstanding, because the correct estimation of ploidy is not overly sensitive to the error model used, it is important to note that our goal in developing this model was to make it complex enough to ensure accurate results, while keeping it simple enough to allow efficient computation. Complexity of the ploidy estimation algorithm is only marginally increased with the full model—that is, we only need to gather auxiliary information within the neighborhood of each nucleotide. In such context, the more informative error model is nevertheless advantageous.

Analysis of a real switchgrass dataset revealed potential issues with the current reference assembly—namely the fact that several contigs may represent paralogs or anciently duplicated regions, which should ideally be separated. We analyzed a small fraction of the switchgrass genome assembly because these data are not yet openly released for whole genome-scale analyses. Indeed, polyploid datasets are only now being extensively obtained; and we propose ConPADE in anticipation of such datasets becoming more commonly analyzed. Additionally, because this is a novel task, to the best of our knowledge, there are currently no other approaches for solving it.

We also analyzed a fraction of the latest wheat genome assembly, which leverages physical chromosome arm separation to reduce assembly complexity. Results for this haploid assembly provided clues about the annotation of repetitive elements, known or putative, and further provided candidates for the inference of intrachromosomal gene duplication. This scenario illustrates other possible applications for ConPADE. Furthermore, we have shown that application of our method to a mock whole genome shotgun assembly of the polyploid wheat genome would correctly identify the ploidy of almost 93% of the contigs, indicating that most of them could be separated by the genome assembler, with very limited collapse of the three subgenomes. When additional WGS data from polyploid species become available, this method can be more extensively tested and improved as necessary. Particularly, the HiSeq error model can be improved to take into account other sources of information about errors or to represent more complex models, for example the inclusion of interactions between variables, or the use of models other than logistic regression to assign error probabilities.

ConPADE is available as a binary executable at https://github.com/microsoftgenomics. Source code will be available in the near future.

## Methods

### HiSeq Error Model

We downloaded data from six different bacterial organisms from the NCBI Sequence Read Archive, according to the following accession numbers: *Eschericia coli* (SRX131047), *Klebsiella oxytoca* (SRX101577), *Mycobacterium tuberculosis* (SRX084335), *Rhodobacter sphaeroides* (SRX160387), *Staphylococcus aureus* (SRX096307) and *Streptococcus pneumoniae* (SRX110128) (Table 2). These species were chosen to represent different bacterial groups with a range of genomic GC contents, and to meet the following criteria: (1) high coverage obtained from whole genome sequencing with the Illumina HiSeq platform, and (2) availability of a finished reference genome for the corresponding strain with a single contig/scaffold closely representing the entire chromosome. We gave preference to curated reference genomes sequenced through capillary methods, whenever possible.

**Table 2:** Bacterial datasets used to learn the error model.

Species	Strain	Genome size (Mb)	GC content (%)	Coverage (X)
*S*. *aureus*	MRSA252	2.90	32.8	1,096.43
*S*. *pneumoniae*	Tigr4	2.16	39.7	533.43
*E*. *coli*	K-12 sub. MG1655	4.64	50.8	239.63
*K*. *oxytoca*	10–5248	6.03^a^	55.0	122.59
*M*. *tuberculosis*	H37Rv	4.41	65.6	633.32^b^
*R*. *sphaeroides*	2.4.1	3.19+0.94	68.8	234.80

^a^For *K*. *oxytoca*, only the largest contig was used, representing approximately 96.95% of the genome.

^b^For *M*. *tuberculosis*, we sampled a small portion of the data to avoid oversampling a single genome (original coverage for downloaded data was 5,598.69X).

We downloaded reference genomes from the NCBI genome archive according to the following assembly numbers: ASM584v1 (*E*. *coli*), PB_Kleb_oxyt_10–5248_V1 (*K*. *oxytoca*), ASM19595v1 (*M*. *tuberculosis*), ASM1290v1 (*R*. *sphaeroides*), ASM1150v1 (*S*. *aureus*) and ASM688v1 (*S*. *pneumoniae*). We aligned the reads against the corresponding reference genome with the Scalable Nucleotide Alignment Program (SNAP) [47] using default parameters, which are tuned for short reads: seed size of 20, maximum combined edit distance of 15 for both reads in a pair, with 25 seeds per read and a maximum of 250 hits considered per seed. We only considered uniquely aligned read pairs for which the distance between mates was within the expected library range.

Next we compared the observed and reference nucleotide, assuming all reference genomes contained no errors. Because real variability is rare in monomorphic bacterial strains, differences between an observed and reference nucleotide are likely due to sequencing errors. We also collected a set of informative features from each available nucleotide: (1) the associated quality score, (2) the neighboring quality score—that is, the average of ten adjacent bases, five on each side, (3) whether the nucleotide was preceded by the 2-mer GG, and (4) the specific nucleotide substitution that took place. Previous studies showed that these features provide more information about the occurrence of (systematic) errors beyond the quality score [30,48,49]. We used these data to estimate parameters of our model relating to the probability of there being a sequencing error, that is, parameters associated with arcs pointing to the *Sequencing error* and *Observed nucleotide* variables in the graphical model of Fig 3.

We held the model structure fixed and estimated parameters for two sets of models. First, we fit a logistic regression model to assign an error probability to each nucleotide observation, with input features representing the logarithm of the quality score, the logarithm of the neighboring quality score, and for each of the four possible nucleotides, whether the base at hand was preceded by the 2-mer GG [49]. The model can be represented as follows:

$$P\left(E=1|T=t,GG=gg,QS=qs,NQS=nqs\right)=\frac{1}{1+e^{\alpha_{t,gg,q}+\beta_{t,gg,q}\text{log}\left(qs\right)+\gamma_{t,gg,q}\text{log}\left(nqs\right)}},$$

where *E* = 1 represents the event of a sequencing error; *T* represents the true nucleotide, with *t* = {*A*, *C*, *G*, *T*}; *GG* is an indicator variable taking value 1 if the nucleotide is preceded by GG and 0 otherwise; *QS* represents the Phred quality score; *NQS* is the average neighboring quality score (*i*.*e*., the average of the 10 closest bases, five on each side); *α*_*t*,*gg*,*q*_, *β*_*t*,*gg*,*q*_ and *γ*_*t*,*gg*,*q*_ are parameters of the model, with *q* an indicator variable taking value 1 if the sequenced base at hand has a quality score of 2 and 0 otherwise. Parameters *β*_*t*,*gg*,1_ were fixed at zero, to take into account the fact that nucleotides with quality 2 deviate from the general trend. We used the scikit-learn Python package to train this model [50]. We applied a 4-fold cross validation scheme to compare this model with the naïve error model that only uses the Phred quality score and sets the probability of observing an error to be *P*(*E* = 1|*QS* = *qs*) = 10^(−*qs*/10)^.

Second, we also used the data to learn specific substitution rates—that is, to estimate probabilities that a given nucleotide was replaced by another specific one. To that end, we estimated multinomial probabilities to represent specific substitution rates, both for cases where the nucleotide was preceded by GG or not. We employed a 10-fold cross validation step to evaluate the fit of the model.

### Ploidy Estimation Model

The likelihood of the model depicted in Fig 3 is given by:

$$\begin{array}{l} L\left(M=m|D\right)\propto P\left(D|M=m\right)\\ =\prod_{p=1}^{C}\sum_{g_{p}=0}^{m}P\left(G_{p}=g_{p}|M=m\right)\\ \;\prod_{i_{p}=1}^{n_{p}}\sum_{t_{i_{p}}=1}^{2}P\left(T_{i_{p}}=t_{i_{p}}|M=m,G_{p}=g_{p}\right)P\left(GG_{i_{p}}=gg_{i_{p}}\right)P\left(QS_{i_{p}}=qs_{i_{p}}\right)P\left(NQS_{i_{p}}=nqs_{i_{p}}\right)\\ \;\sum_{e_{i_{p}}=0}^{1}P\left(E_{i_{p}}=e_{i_{p}}|T_{i_{p}}=t_{i_{p}},GG_{i_{p}}=gg_{i_{p}},QS_{i_{p}}=qs_{i_{p}},NQS_{i_{p}}=nqs_{i_{p}}\right)\\ \;P\left(O_{i_{p}}=o_{i_{p}}|T_{i_{p}}=t_{i_{p}},E_{i_{p}}=e_{i_{p}},GG_{i_{p}}=gg_{i_{p}}\right) \end{array}$$

where *p* = 1,⋯, *C* corresponds to a position in a contig of length *C*, *i*_*p*_ = 1,⋯, *n*_*p*_ is the *i*th read covering position *p* and *n*_*p*_ is the total number of reads covering the same position, $GG_{i_{p}}$ is an observed variable indicating whether a nucleotide is preceded by GG, $QS_{i_{p}}$ is the associated quality score; $NQS_{i_{p}}$ is the neighboring quality score (*i*.*e*., the average of the 10 closest bases, five on each side), $E_{i_{p}}$ represents whether the current base is a sequencing error or not, $O_{i_{p}}$ denotes the observed nucleotide for read *i* in position *p*, and other variables are as previously defined. Because many variables are always observed, this expression can be simplified to:

$$\begin{array}{l} L\left(M=m|D\right)\propto \\ =\prod_{p=1}^{C}\sum_{g_{p}=0}^{m}P\left(G_{p}=g_{p}|M=m\right)\prod_{i_{p}=1}^{n_{p}}\sum_{t_{i_{p}}=1}^{2}P\left(T_{i_{p}}=t_{i_{p}}|M=m,G_{p}=g_{p}\right)\\ \;\sum_{e_{i_{p}}=0}^{1}P\left(E_{i_{p}}=e_{i_{p}}|T_{i_{p}}=t_{i_{p}},GG_{i_{p}}=gg_{i_{p}},QS_{i_{p}}=qs_{i_{p}},NQS_{i_{p}}=nqs_{i_{p}}\right)\\ \;P\left(O_{i_{p}}=o_{i_{p}}|T_{i_{p}}=t_{i_{p}},E_{i_{p}}=e_{i_{p}},GG_{i_{p}}=gg_{i_{p}}\right) \end{array}$$

As most variables are observed, marginalization has to be done only for the unknown true nucleotide (variable $T_{i_{p}}$) and the possible occurrence of a sequencing error ($E_{i_{p}}$), which makes such inference efficient for scaffolds millions of nucleotides long covered at high sequencing depth. The remaining summation and product terms gather information from all reads in all positions, for all possible genotypes under a given ploidy. The above computations are done for the desired range of ploidies. The ploidy with the maximum likelihood is chosen.

## Simulations

For the first set of simulations, we created a 10 Mb long consensus sequence for each ploidy level, from one to 16. We then simulated heterozygous sites at an average interval of 1,000 bases, and for each SNP we uniformly sampled the dosage from 1 to (ploidy−1). Illumina short reads were simulated through SimSeq [51], in pairs of 100 bases, at different levels of coverage: 10X, 15X, 25X, 50X and 75X per haploid unit (*i*.*e*., per contig copy). SimSeq uses real Illumina runs to generate empirical short read profiles including sequencing errors and quality scores. We note that such sequencing runs are completely independent from the ones we used to train our error model. We aligned the reads against the corresponding scaffold with SNAP and applied our method to estimate ploidy and call variants for each scenario. We conducted dosage analyses only for cases where the correct ploidy was estimated and employed a Phred-like threshold of 40 to call variants.

To further evaluate the effect of sequencing coverage on ploidy and dosage estimation, we subsequently simulated 100 sets of consensus contigs with 200 kb in length each, for ploidy levels again ranging from one to 16. We simulated variant positions and dosages using the same criteria as before, and simulated sequencing reads at coverage levels of 10X, 15X, 25X and 50X. Analysis of each simulated contig followed the same approach as for the previous scenario, comprising read alignment, ploidy estimation, variant calling and dosage inference.

Next, to evaluate the effect of contig length on ploidy estimation, we simulated contigs with lengths 2,000, 20,000 and 200,000 bases, for each ploidy. We simulated one SNP every 200 bases, such that the number of informative variants was on average 10, 100 and 1,000, respectively, for the different contig lengths. We employed the same uniform distribution to simulate allele dosages. Finally, we simulated short reads at 50X coverage per haploid copy. We simulated each combination of ploidy and contig length 100 times to provide an estimate of ploidy estimation accuracy. Again, for each simulation, we aligned reads against the original contig and applied ConPADE.

We also analyzed all simulated datasets with the ploidy estimation model, but replacing our calculated HiSeq error model with the naïve model that only takes quality scores into account. Our goal is not in exhaustively comparing both error models, but rather assessing how strongly the error model affects ploidy estimates and variant dosage calls.

### Analysis of a Switchgrass Dataset

We downloaded the *Panicum virgatum* AP13 genome reference from Phytozome (http://www.phytozome.com/panicumvirgatum.php). For all analyses, we utilized the genomic assembly hardmasked for repetitive sequence, to ensure spurious alignments to repetitive regions did not affect results. We also downloaded from the NCBI Sequence Read Archive whole genome shotgun reads from AP13, from accession numbers SRX109496, SRX109498, SRX109499, SRX109501, SRX109503, SRX109505, SRX110233 and SRX110234. We only used the first run for the latter accession. There were a total of 106.4 Gb of sequence in 354,733,809 read pairs of 150 nucleotides each, 102 Gb in 340,008,647 read pairs of 157 and 143 nucleotides, and 103 Gb in 515,426,302 read pairs of 100 nucleotides each.

Next we then aligned all read pairs against the reference genome with Bowtie 2 [52], using very sensitive parameters. If the distance between paired reads was outside the expected fragment size, the Phred mapping quality was below 40, or the reads were marked as PCR duplicates, the read pair was discarded. Finally, we randomly sampled 5,000 contigs and used the alignment results as input for the ploidy estimation method and for variant calling. For this analysis, we set *P*(*SNP*) equal to one variant every 200 bases, to represent SNP densities commonly observed in higher plants [53–55]. We estimated the most likely ploidy for each sampled contig and calculated SNP posterior probabilities, which were subsequently used for variant calling. The existence of variants at positions with Phred score over 40 were deemed significant.

### Analysis of a Wheat Dataset

We downloaded the survey sequence assembly of the large arm of chromosomes 5A, 5B and 5D from *Triticum aestivum* L., genotype Chinese Spring line 42 (CS42) (http://urgi.versailles.inra.fr/download/iwgsc/). Next we downloaded from the EMBL-EBI European Nucleotide Archive shotgun reads from the same chromosome arms, also from genotype CS42, obtained through the Illumina Genome Analyzer IIx and HiSeq 2000 technologies, corresponding to accession numbers ERR277132 through ERR277135 (large arm of chromosome 5A), ERR277139 and ERR277140 (large arm of chromosome 5B), and ERR277146 and ERR277147 (large arm of chromosome 5D). These runs comprised 25.72 Gb, 60.58 Gb and 46.26 Gb of sequence for chromosomes 5A, 5B and 5D, respectively. Based on the estimated wheat chromosome arm sizes [56], these data correspond to coverage levels of 48.35X, 104.45X and 94.41X, for chromosomes 5A, 5B and 5D, respectively.

As an initial validation procedure, we aligned all read pairs from chromosome 5D against its corresponding reference arm assembly with Bowtie 2 [52], using very sensitive parameters. We only kept reads for which the distance between mates was within the empirically determined library fragment length distribution, and for which the Phred mapping quality was higher than 40. Reads marked as PCR duplicates were removed from the analysis. We used these alignment results as input for the ploidy estimation method and for variant calling, again setting *P*(*SNP*) to one variant every 200 bases. Ploidy estimation and variant calling followed the same strategy as for the switchgrass dataset.

After ploidy estimation analyses, we sampled 30 contigs with a ploidy estimate of four and conducted manual annotation via BLAST searches [41]. To that end, we aligned these contigs to the NCBI nucleotide (NT) database with BLASTN, using default parameters. We filtered alignments with an E-value cutoff of 1*e* ^−10^ and manually parsed the results. When a contig aligned to a BAC or other long sequence with multiple annotations, we only considered the portion to which said contig actually aligned.

Next, we performed similar analyses for a dataset obtained by pooling data from the three individual wheat subgenomes. To that end, we firstly downsampled data from chromosomes 5B and 5D, such that the coverage levels for the three subgenomes were equivalent. We then assembled the combined reads with ABySS [11], using a k-mer size of 71 to mirror the strategy utilized for the assembly of the wheat genome [40]. In order to assess the presence or absence of each resulting contig in the original subgenome assemblies, we aligned the contigs to the individual assemblies using BLAST [41], and considered a contig to be present in a given subgenome if an alignment to a reference contig covered more than 95% of the query length with more than 95% identity. Finally, we aligned the pooled short reads to our newly created assembly and conducted ploidy estimation using the same criteria applied to the individual analysis of the large arm of chromosome 5D. We supplemented these analyses by estimating the ploidy of each contig in each individual subgenome, by separately aligning reads from the three subgenomes and applying ConPADE.

## Supporting Information

S1 Fig Distribution of Phred quality scores for combined bacterial datasets. (TIF)

S2 Fig Coverage simulation results. A white cell indicates an error in ploidy estimation, with the corresponding called ploidy overlaid. Color in each cell indicates the percentage of correct variant dosage calls for scenarios where ConPADE identified the correct ploidy.
(TIF)

S3 Fig Ploidy calls for 100 contigs with different simulated ploidies and sequencing coverage of 15X. Each panel represents the distribution of ploidy calls from 100 contigs, 200 kb in length each, with a given simulated ploidy level. Ploidy calls made with the full error model.
(TIF)

S4 Fig Allele dosage calls of SNPs in contigs with ploidy 15 and coverage 15X. Each panel represents the distribution of ConPADE dosage calls from a set of variants with a given simulated allele dosage. Data from 33 contigs with a correctly estimated ploidy of 15, with 200 SNPs each. Only significantly called SNPs included. Dosage calls made with the full error model.
(TIF)

S5 Fig Ploidy estimation accuracy for varying contig lengths and coverage levels. Accuracy indicates the number of correct calls out of 100 simulations. Ploidy calls made with the full error model.
(TIF)

S6 Fig Distribution of contig lengths from the switchgrass genome assembly. Only contigs shorter than 30 kb are shown.
(TIF)

S7 Fig Stack of short reads aligned against a sub-region of switchgrass contig 17625. (TIF)

S1 Table Coverage simulation results. Top (bottom) number in each cell represents the most likely ploidy estimated for a 10 Mb-long contig with the full (naïve) error model. Corresponding percentages of correct variant dosage calls are inside parentheses. Variant calling accuracy was not measured when the ploidy was incorrectly estimated.
(DOCX)

S2 Table Results from the coverage simulations. Top (bottom) number in each cell displays the results with the full (naïve) error model, out of 100 simulations of 200 kb-long contigs for each scenario. FNR denotes false negative rate of SNP detection.
(DOCX)

S3 Table Results of ploidy calls for a simulated coverage of 15X. The number in each cell represents the frequency of each estimated ploidy for a set of 100 contigs, 200 kb in length each, for each simulated ploidy level. Ploidy calls made with the full error model.
(DOCX)

S4 Table Results of SNP dosage calls for a simulated ploidy of 15 and coverage of 15X. The number in each cell represents the frequency of each estimated dosage for a set of SNPs from 33 contigs, with 200 SNPs each, for each simulated dosage level. Only cases in which the estimated ploidy was correct and the SNP was deemed significant are included. Dosage calls made with the full error model.
(DOCX)

S5 Table Results from the length simulations. Top (bottom) number in each cell displays the results with the full (naïve) error model, out of 100 simulations for each scenario, each with 50X coverage. FNR denotes false negative rate of SNP detection.
(DOCX)

S6 Table Wheat annotation results. Annotation results of 30 wheat (*Triticum aestivum*) contigs from chromosome arm 5D with an estimated ploidy of four. Results based on BLASTN alignments against the nucleotide database of NCBI (NT).
(DOCX)
