# Supplementary figures and images for: ConPADE: Genome Assembly Ploidy Estimation from Next-Generation Sequencing Data
Source: PLoS Comput Biol. 2015 Apr 16;11(4):e1004229. doi: 10.1371/journal.pcbi.1004229 (PMC4400156; doi:10.1371/journal.pcbi.1004229)

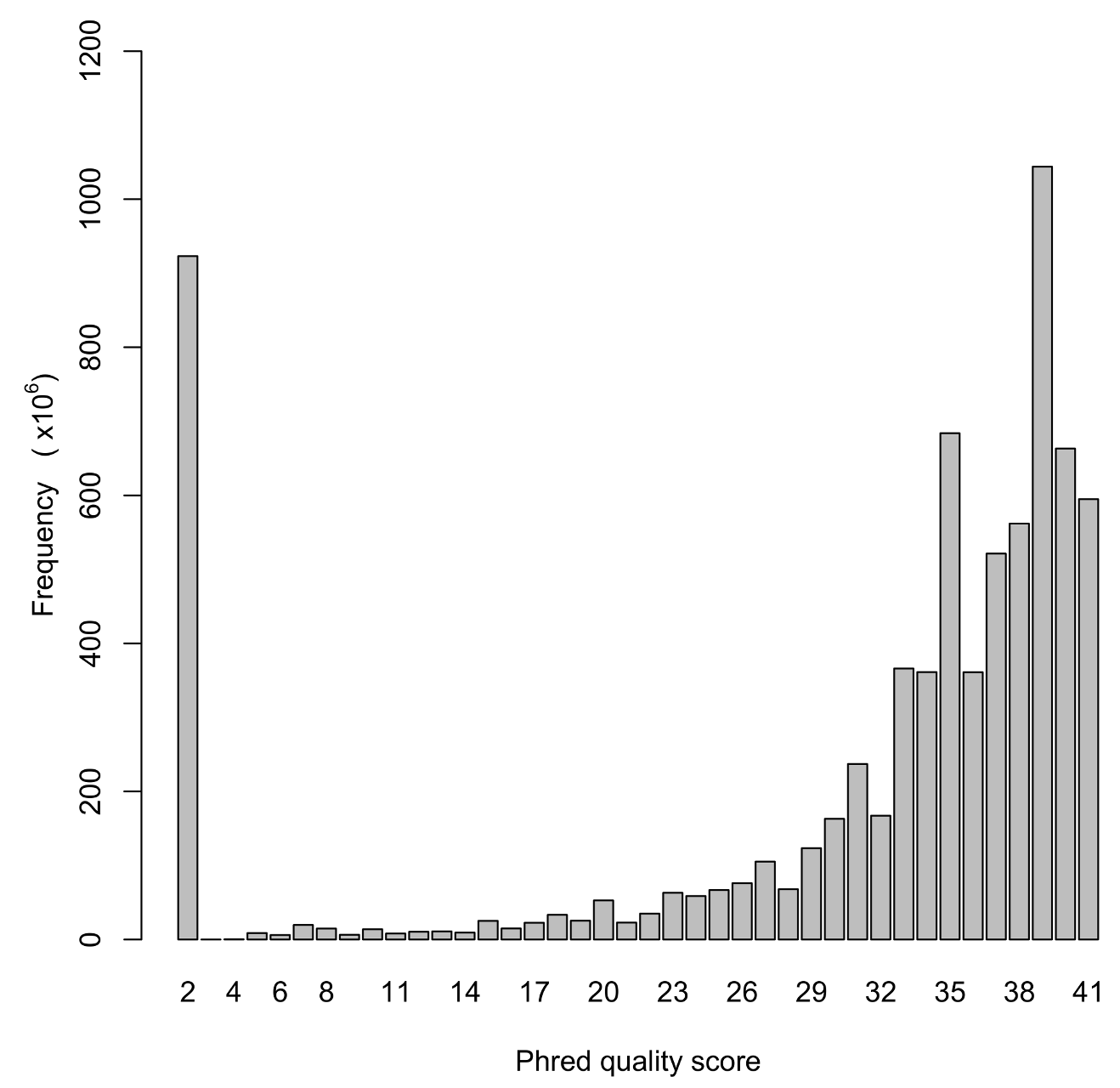

Supplement: S1 Fig — (TIF) [file pcbi.1004229.s001.tif]

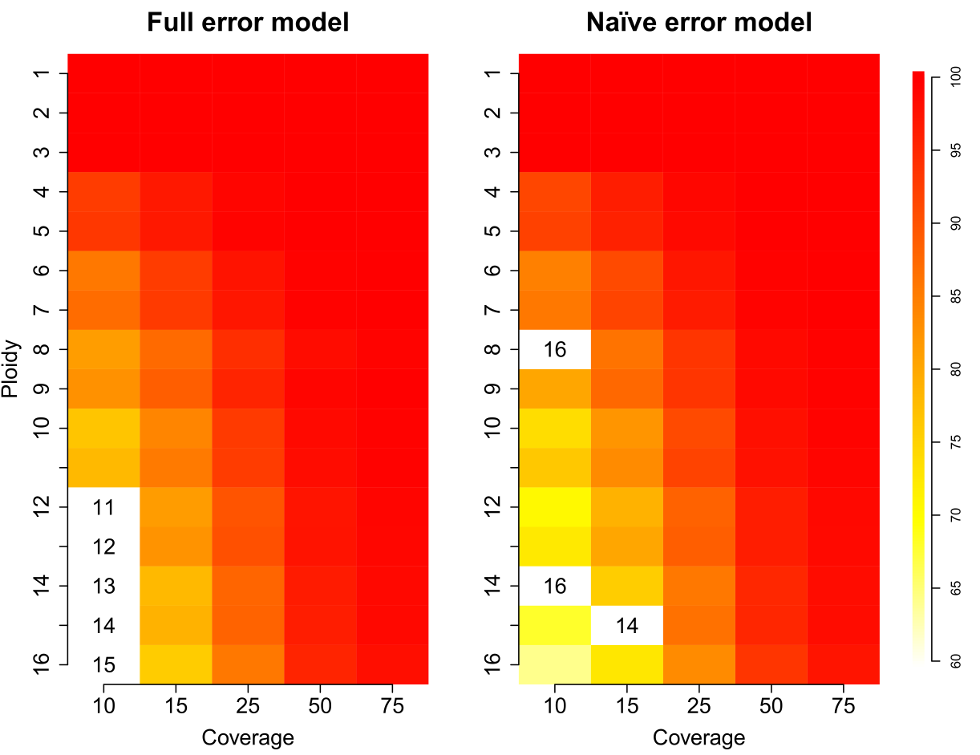

Supplement: S2 Fig — A white cell indicates an error in ploidy estimation, with the corresponding called ploidy overlaid. Color in each cell indicates the percentage of correct variant dosage calls for scenarios where ConPADE identified the correct ploidy. (TIF) [file pcbi.1004229.s002.tif]

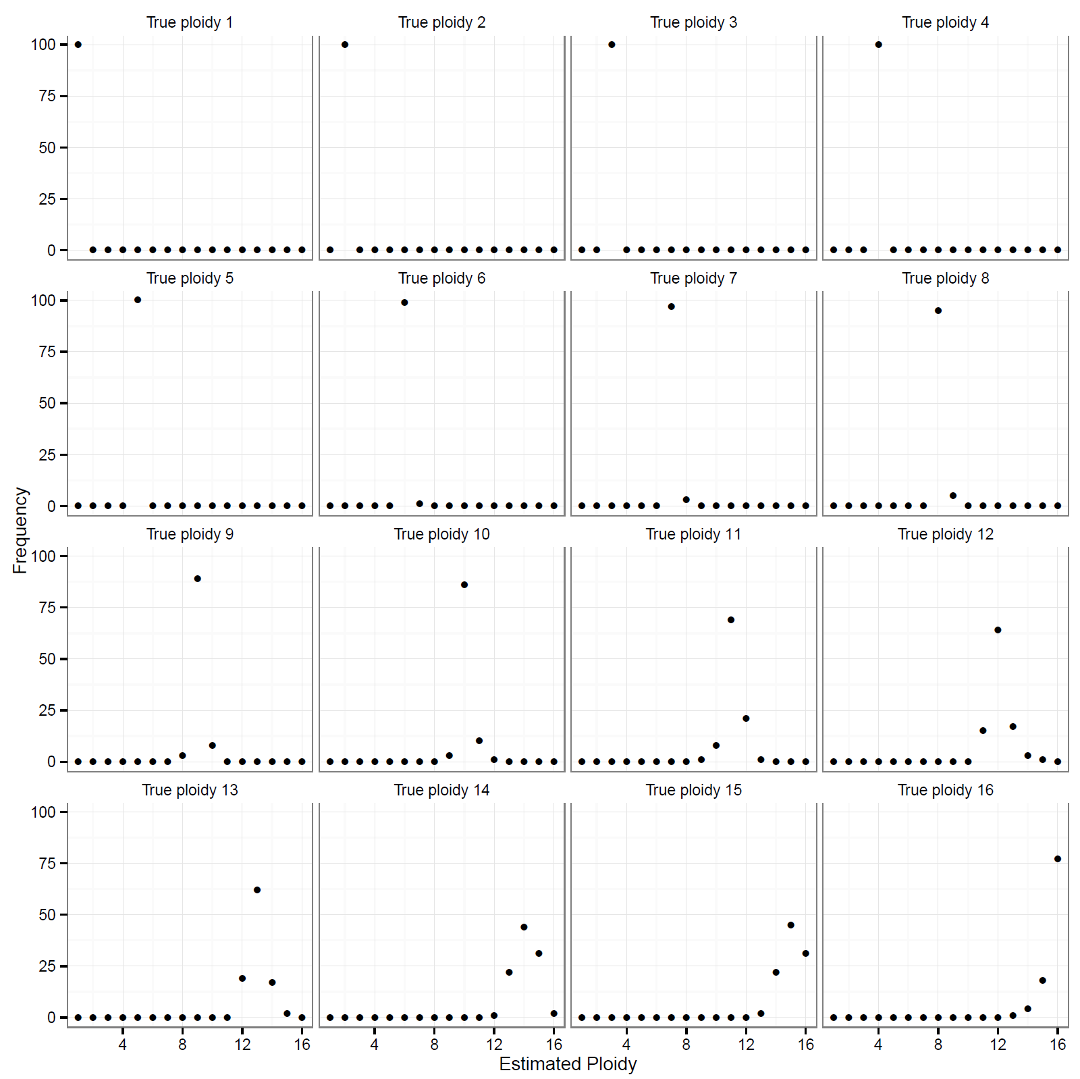

Supplement: S3 Fig — Each panel represents the distribution of ploidy calls from 100 contigs, 200 kb in length each, with a given simulated ploidy level. Ploidy calls made with the full error model. (TIF) [file pcbi.1004229.s003.tif]

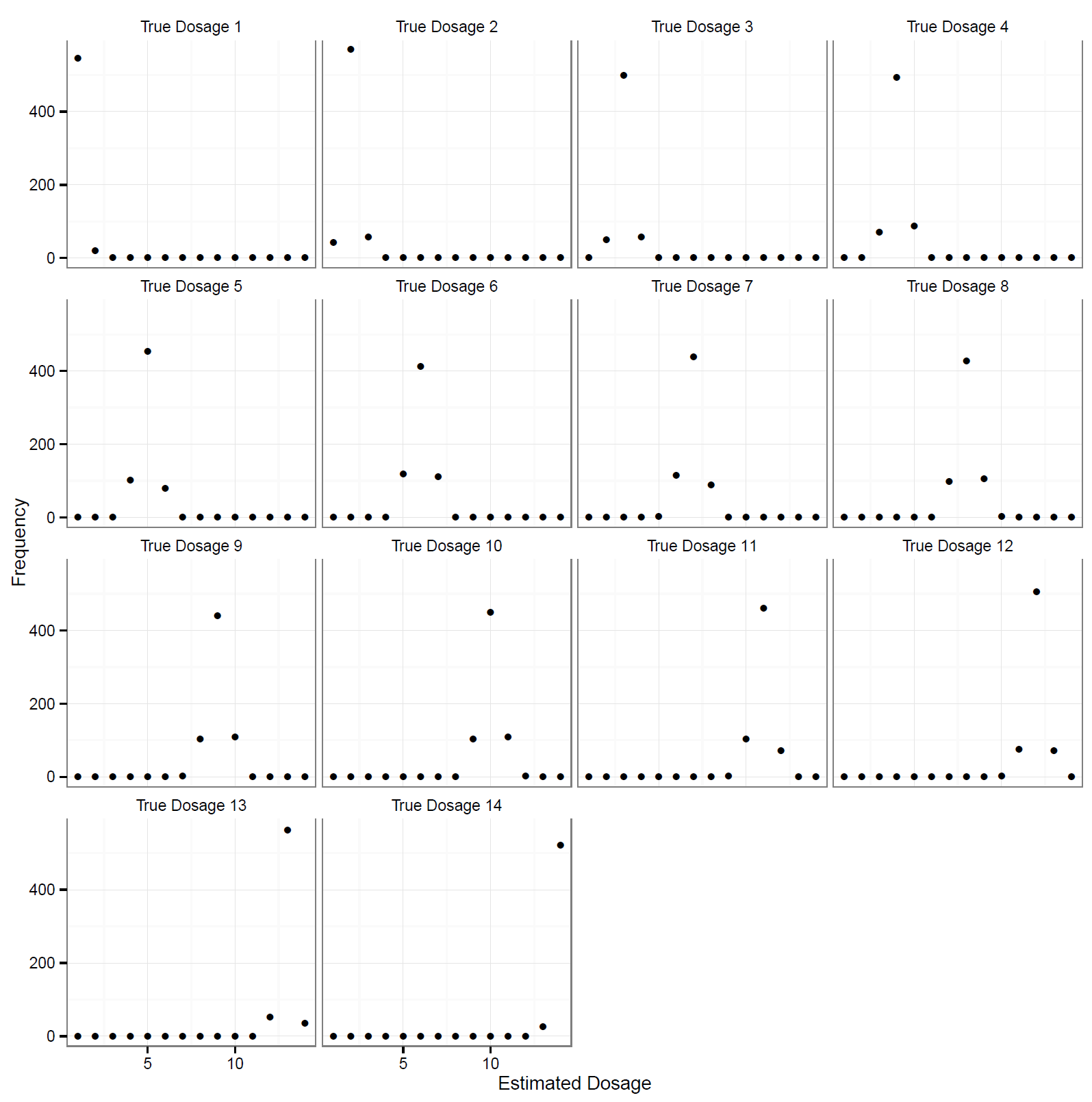

Supplement: S4 Fig — Each panel represents the distribution of ConPADE dosage calls from a set of variants with a given simulated allele dosage. Data from 33 contigs with a correctly estimated ploidy of 15, with 200 SNPs each. Only significantly called SNPs included. Dosage calls made with the full error model. (TIF) [file pcbi.1004229.s004.tif]

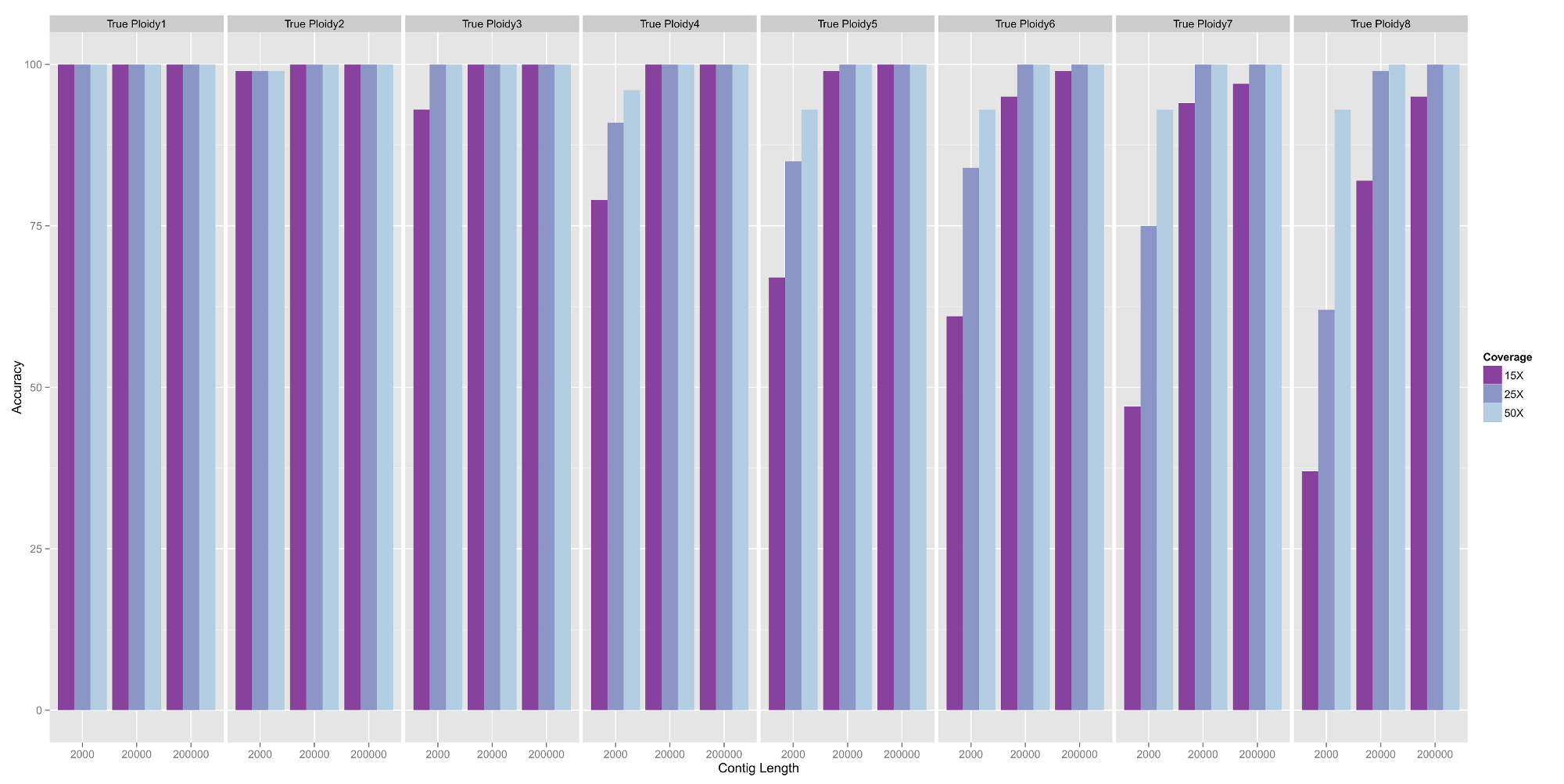

Supplement: S5 Fig — Accuracy indicates the number of correct calls out of 100 simulations. Ploidy calls made with the full error model. (TIF) [file pcbi.1004229.s005.tif]

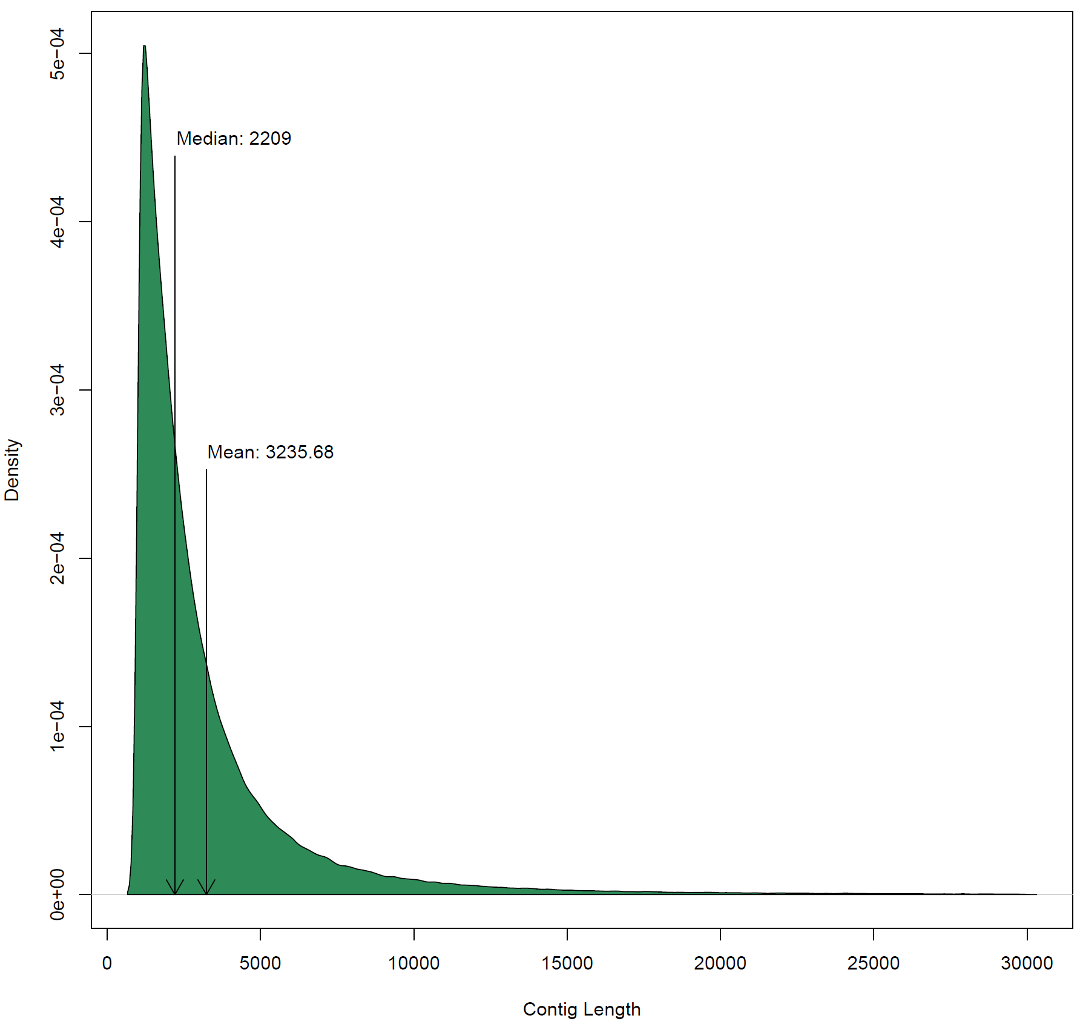

Supplement: S6 Fig — Only contigs shorter than 30 kb are shown. (TIF) [file pcbi.1004229.s006.tif]

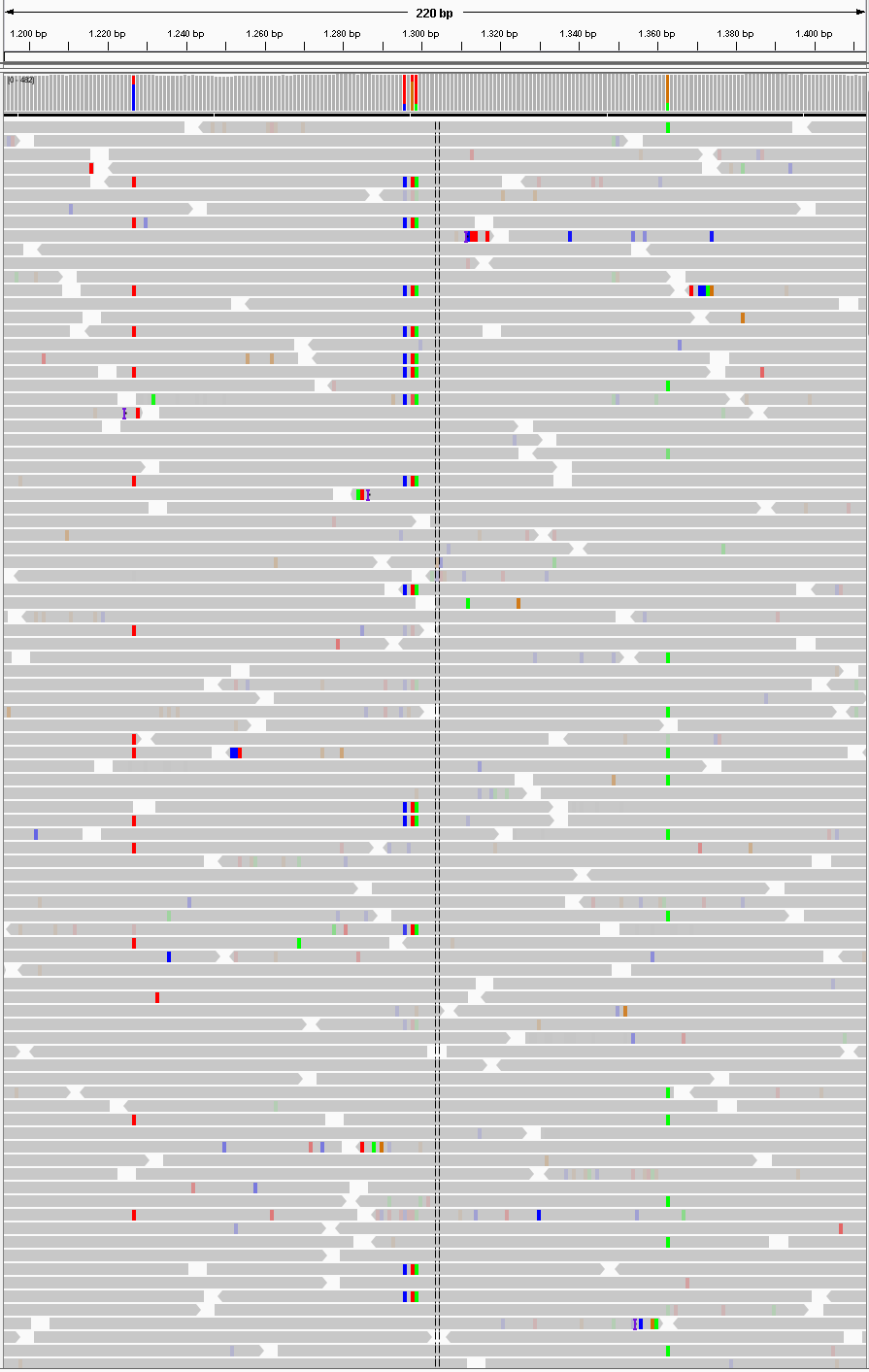

Supplement: S7 Fig — (TIF) [file pcbi.1004229.s007.tif]
