# Supplementary material for: ConPADE: Genome Assembly Ploidy Estimation from Next-Generation Sequencing Data
Source: PLoS Comput Biol. 2015 Apr 16;11(4):e1004229. doi: 10.1371/journal.pcbi.1004229 (PMC4400156; doi:10.1371/journal.pcbi.1004229)
Supplement: S1 Table — Top (bottom) number in each cell represents the most likely ploidy estimated for a 10 Mb-long contig with the full (naïve) error model. Corresponding percentages of correct variant dosage calls are inside parentheses. Variant calling accuracy was not measured when the ploidy was incorrectly estimated. (DOCX) [file pcbi.1004229.s008.docx]

**S1 Table:** Coverage simulation results. Top (bottom) number in each cell represents the most likely ploidy estimated for a 10 Mb-long contig with the full (naïve) error model. Corresponding percentages of correct variant dosage calls are inside parentheses. Variant calling accuracy was not measured when the ploidy was incorrectly estimated.

| True Ploidy | Coverage per haploid copy | | | | |
| --- | --- | --- | --- | --- | --- |
|  | 10X | 15X | 25X | 50X | 75X |
| 1 | 1  1 | 1  1 | 1  1 | 1  1 | 1  1 |
| 2 | 2 (100%)  2 (100%) | 2 (100%)  2 (100%) | 2 (100%)  2 (100%) | 2 (100%)  2 (100%) | 2 (100%)  2 (100%) |
| 3 | 3 (100%)  3 (100%) | 3 (100%)  3 (100%) | 3 (100%)  3 (100%) | 3 (100%)  3 (100%) | 3 (100%)  3 (100%) |
| 4 | 4 (92.98%)  4 (91.73%) | 4 (97.03%)  4 (96.43%) | 4 (99.40%)  4 (99.03%) | 4 (99.97%)  4 (99.96%) | 4 (100%)  4 (100%) |
| 5 | 5 (93.56%)  5 (92.38%) | 5 (97.02%)  5 (96.06%) | 5 (99.42%)  5 (98.70%) | 5 (99.96%)  5 (99.93%) | 5 (99.99%)  5 (99.98%) |
| 6 | 6 (86.00%)  6 (84.94%) | 6 (92.97%)  6 (91.28%) | 6 (97.78%)  6 (97.23%) | 6 (99.80%)  6 (99.65%) | 6 (99.99%)  6 (99.98%) |
| 7 | 7 (87.34%)  7 (85.83%) | 7 (93.02%)  7 (91.88%) | 7 (97.37%)  7 (96.63%) | 7 (99.77%)  7 (99.54%) | 7 (99.97%)  7 (99.94%) |
| 8 | 8 (81.48%)  16 | 8 (87.44%)  8 (86.46%) | 8 (94.47%)  8 (93.66%) | 8 (98.58%)  8 (98.80%) | 8 (99.49%)  8 (99.63%) |
| 9 | 9 (82.94%)  9 (80.56%) | 9 (88.90%)  9 (87.75%) | 9 (95.79%)  9 (93.72%) | 9 (99.34%)  9 (98.79%) | 9 (99.89%)  9 (99.72%) |
| 10 | 10 (76.87%)  10 (74.01%) | 10 (84.38%)  10 (82.41%) | 10 (93.11%)  10 (91.32%) | 10 (98.79%)  10 (98.14%) | 10 (99.73%)  10 (99.49%) |
| 11 | 11 (78.28%)  11 (76.44%) | 11 (85.70%)  11 (83.80%) | 11 (92.86%)  11 (92.03%) | 11 (98.44%)  11 (97.81%) | 11 (99.68%)  11 (99.35%) |
| 12 | 11  12 (70.93%) | 12 (81.78%)  12 (79.08%) | 12 (90.17%)  12 (88.47%) | 12 (97.53%)  12 (96.59%) | 12 (99.28%)  12 (98.95%) |
| 13 | 12  13 (72.65%) | 13 (82.64%)  13 (80.48%) | 13 (90.49%)  13 (88.93%) | 13 (97.68%)  13 (96.44%) | 13 (99.20%)  13 (98.75%) |
| 14 | 13  16 | 14 (78.27%)  14 (75.98%) | 14 (88.04%)  14 (85.97%) | 14 (96.70%)  14 (95.38%) | 14 (98.98%)  14 (98.48%) |
| 15 | 14  15 (68.39%) | 15 (79.15%)  14 | 15 (88.28%)  15 (86.69%) | 15 (96.44%)  15 (95.44%) | 15 (98.88%)  15 (98.41%) |
| 16 | 15  16 (64.44%) | 16 (76.12%)  16 (72.88%) | 16 (85.92%)  16 (83.79%) | 16 (95.61%)  16 (93.70%) | 16 (98.39%)  16 (97.59%) |
