# Supplementary material for: ConPADE: Genome Assembly Ploidy Estimation from Next-Generation Sequencing Data
Source: PLoS Comput Biol. 2015 Apr 16;11(4):e1004229. doi: 10.1371/journal.pcbi.1004229 (PMC4400156; doi:10.1371/journal.pcbi.1004229)
Supplement: S2 Table — Top (bottom) number in each cell displays the results with the full (naïve) error model, out of 100 simulations of 200 kb-long contigs for each scenario. FNR denotes false negative rate of SNP detection. (DOCX) [file pcbi.1004229.s009.docx]

**S2 Table:** Results from the coverage simulations. Top (bottom) number in each cell displays the results with the full (naïve) error model, out of 100 simulations of 200 kb-long contigs for each scenario. FNR denotes false negative rate of SNP detection.

| True Ploidy | Coverage | | | | | | | | | | | | | | |
| --- | --- | --- | --- | --- | --- | --- | --- | --- | --- | --- | --- | --- | --- | --- | --- |
|  | 10X | | |  | 15X | | |  | 25X | | |  | 50X | | |
|  | Correct ploidy (%) | Correct dosage (%) | FNR (%) |  | Correct ploidy (%) | Correct dosage (%) | FNR (%) |  | Correct ploidy (%) | Correct dosage (%) | FNR (%) |  | Correct ploidy (%) | Correct dosage (%) | FNR (%) |
| 1 | 100  100 | --- | --- |  | 100  100 | --- | --- |  | 100  100 | --- | --- |  | 100  100 | --- | --- |
| 2 | 100  100 | 100  100 | 7.05  3.39 |  | 100  100 | 100  100 | 0.72  0.41 |  | 100  100 | 100  100 | 0.06  0.05 |  | 100  100 | 100  100 | 0.03  0.03 |
| 3 | 100  99 | 100  100 | 6.13  2.54 |  | 100  100 | 100  100 | 0.80  0.23 |  | 100  100 | 100  100 | 0.03  0.02 |  | 100  100 | 100  100 | 0.02  0.02 |
| 4 | 100  86 | 92.35  91.55 | 6.38 2.20 |  | 100  100 | 96.85  95.76 | 0.85  0.18 |  | 100  100 | 99.30  98.91 | 0.03  0.02 |  | 100  100 | 99.87  99.77 | 0.03  0.02 |
| 5 | 99  72 | 93.35  92.79 | 6.09  1.83 |  | 100  100 | 96.71  95.83 | 1.09  0.20 |  | 100  100 | 99.09  98.58 | 0.04  0.02 |  | 100  100 | 99.89  99.75 | 0.02  0.02 |
| 6 | 95  56 | 85.71  84.18 | 6.40  2.05 |  | 99  99 | 92.14  90.75 | 1.20  0.26 |  | 100  100 | 97.25  96.41 | 0.08  0.02 |  | 100  100 | 99.36  99.04 | 0.02  0.02 |
| 7 | 82  48 | 87.03  85.87 | 6.17  1.91 |  | 97  96 | 92.66  91.54 | 1.38  0.31 |  | 100  100 | 97.35  96.50 | 0.05  0.02 |  | 100  100 | 99.35  99.04 | 0.02  0.00 |
| 8 | 81  38 | 80.27 79.26 | 5.97  1.68 |  | 95  89 | 88.35  86.71 | 1.35  0.20 |  | 100  100 | 95.17  93.75 | 0.06  0.00 |  | 100  100 | 98.51  97.73 | 0.02  0.01 |
| 9 | 61  33 | 82.02  80.67 | 6.11  1.75 |  | 89  74 | 89.48  88.03 | 1.28  0.25 |  | 100  99 | 95.55  94.05 | 0.08  0.01 |  | 100  100 | 99.21  98.71 | 0.01  0.01 |
| 10 | 50  32 | 75.6  74.06 | 5.95  1.70 |  | 86  74 | 85.37  83.33 | 1.40  0.24 |  | 99  100 | 92.82  91.21 | 0.11  0.03 |  | 100  100 | 98.85  98.16 | 0.02  0.01 |
| 11 | 43  23 | 77.4  76.15 | 5.93  1.61 |  | 69  56 | 85.58  83.58 | 1.45  0.26 |  | 98  97 | 93.05  91.31 | 0.12  0.03 |  | 100  100 | 98.55  97.62 | 0.01  0.01 |
| 12 | 37  21 | 71.29  69.62 | 6.10  1.61 |  | 64  44 | 81.13  80.22 | 1.74  0.24 |  | 95  90 | 90.52  88.60 | 0.10  0.02 |  | 100  100 | 97.88  96.85 | 0.02  0.02 |
| 13 | 33  21 | 73.00  71.78 | 5.98  1.66 |  | 62  40 | 81.62  79.40 | 1.73  0.27 |  | 91  88 | 90.64  88.59 | 0.15  0.02 |  | 100  99 | 95.67  94.27 | 0.02  0.02 |
| 14 | 20  15 | 67.59  65.9 | 5.61  1.60 |  | 44  44 | 78.17  76.71 | 1.80  0.22 |  | 83  77 | 88.06  86.12 | 0.11  0.02 |  | 100  96 | 94.45  92.85 | 0.01  0.01 |
| 15 | 19  16 | 69.78  68.86 | 5.94  1.69 |  | 45  33 | 78.79  77.05 | 1.98  0.30 |  | 83  63 | 88.92  86.82 | 0.22 0.03 |  | 100  96 | 96.68  95.44 | 0.02  0.01 |
| 16 | 24  71 | 64.32  63.88 | 5.95  1.72 |  | 77  54 | 75.56  72.73 | 2.02  0.31 |  | 98  55 | 86.08  83.92 | 0.18  0.02 |  | 100  96 | 95.34  93.59 | 0.00  0.00 |
