# Supplementary material for: ConPADE: Genome Assembly Ploidy Estimation from Next-Generation Sequencing Data
Source: PLoS Comput Biol. 2015 Apr 16;11(4):e1004229. doi: 10.1371/journal.pcbi.1004229 (PMC4400156; doi:10.1371/journal.pcbi.1004229)
Supplement: S4 Table — The number in each cell represents the frequency of each estimated dosage for a set of SNPs from 33 contigs, with 200 SNPs each, for each simulated dosage level. Only cases in which the estimated ploidy was correct and the SNP was deemed significant are included. Dosage calls made with the full error model. (DOCX) [file pcbi.1004229.s011.docx]

**S4 Table:** Results of SNP dosage calls for a simulated ploidy of 15 and coverage of 15X. The number in each cell represents the frequency of each estimated dosage for a set of SNPs from 33 contigs, with 200 SNPs each, for each simulated dosage level. Only cases in which the estimated ploidy was correct and the SNP was deemed significant are included. Dosage calls made with the full error model.

| True Dosage | Estimated Dosage | | | | | | | | | | | | | |
| --- | --- | --- | --- | --- | --- | --- | --- | --- | --- | --- | --- | --- | --- | --- |
|  | 1 | 2 | 3 | 4 | 5 | 6 | 7 | 8 | 9 | 10 | 11 | 12 | 13 | 14 |
| 1 | 546 | 20 | 0 | 0 | 0 | 0 | 0 | 0 | 0 | 0 | 0 | 0 | 0 | 0 |
| 2 | 42 | 570 | 57 | 0 | 0 | 0 | 0 | 0 | 0 | 0 | 0 | 0 | 0 | 0 |
| 3 | 0 | 49 | 499 | 57 | 0 | 0 | 0 | 0 | 0 | 0 | 0 | 0 | 0 | 0 |
| 4 | 0 | 0 | 70 | 492 | 87 | 0 | 0 | 0 | 0 | 0 | 0 | 0 | 0 | 0 |
| 5 | 0 | 0 | 1 | 101 | 453 | 78 | 1 | 0 | 0 | 0 | 0 | 0 | 0 | 0 |
| 6 | 0 | 0 | 0 | 0 | 118 | 412 | 110 | 0 | 0 | 0 | 0 | 0 | 0 | 0 |
| 7 | 0 | 0 | 0 | 0 | 2 | 115 | 438 | 88 | 0 | 0 | 0 | 0 | 0 | 0 |
| 8 | 0 | 0 | 0 | 0 | 0 | 1 | 97 | 427 | 105 | 2 | 0 | 0 | 0 | 0 |
| 9 | 0 | 0 | 0 | 0 | 0 | 0 | 2 | 103 | 440 | 108 | 0 | 0 | 0 | 0 |
| 10 | 0 | 0 | 0 | 0 | 0 | 0 | 0 | 0 | 103 | 449 | 108 | 1 | 0 | 0 |
| 11 | 0 | 0 | 0 | 0 | 0 | 0 | 0 | 0 | 1 | 103 | 460 | 70 | 0 | 0 |
| 12 | 0 | 0 | 0 | 0 | 0 | 0 | 0 | 0 | 0 | 1 | 75 | 505 | 70 | 0 |
| 13 | 0 | 0 | 0 | 0 | 0 | 0 | 0 | 0 | 0 | 0 | 0 | 52 | 562 | 35 |
| 14 | 0 | 0 | 0 | 0 | 0 | 0 | 0 | 0 | 0 | 0 | 0 | 0 | 25 | 521 |
