# Supplementary material for: ConPADE: Genome Assembly Ploidy Estimation from Next-Generation Sequencing Data
Source: PLoS Comput Biol. 2015 Apr 16;11(4):e1004229. doi: 10.1371/journal.pcbi.1004229 (PMC4400156; doi:10.1371/journal.pcbi.1004229)
Supplement: S5 Table — Top (bottom) number in each cell displays the results with the full (naïve) error model, out of 100 simulations for each scenario, each with 50X coverage. FNR denotes false negative rate of SNP detection. (DOCX) [file pcbi.1004229.s012.docx]

**S5 Table:** Results from the length simulations. Top (bottom) number in each cell displays the results with the full (naïve) error model, out of 100 simulations for each scenario, each with 50X coverage. FNR denotes false negative rate of SNP detection.

| True Ploidy | Contig length | | | | | | | | | | |
| --- | --- | --- | --- | --- | --- | --- | --- | --- | --- | --- | --- |
|  | 2000 | | |  | 20000 | | |  | 200000 | | |
|  | Correct ploidy (%) | Correct dosage (%) | FNR (%) |  | Correct ploidy (%) | Correct dosage (%) | FNR (%) |  | Correct ploidy (%) | Correct dosage (%) | FNR (%) |
| 1 | 100  100 | --- | --- |  | 100  100 | --- | --- |  | 100  100 | --- | --- |
| 2 | 99  99 | 100  100 | 3.55  2.90 |  | 100  100 | 100  100 | 0.32  0.28 |  | 100  100 | 100  100 | 0.05  0.04 |
| 3 | 100  100 | 100  100 | 3.41  3.22 |  | 100  100 | 100  100 | 0.37  0.33 |  | 100  100 | 100  100 | 0.05  0.04 |
| 4 | 96  96 | 99.28  99.38 | 2.81  2.31 |  | 100  100 | 99.97  99.94 | 0.29  0.24 |  | 100  100 | 99.98  99.95 | 0.03  0.02 |
| 5 | 93  93 | 99.15  99.37 | 2.88  2.57 |  | 100  100 | 99.91  99.90 | 0.29  0.20 |  | 100  100 | 99.96  99.93 | 0.03  0.02 |
| 6 | 93  94 | 99.55  99.44 | 2.73  2.29 |  | 100  100 | 99.81  99.58 | 0.32  0.28 |  | 100  100 | 99.80  99.67 | 0.04  0.02 |
| 7 | 93  93 | 97.97  97.64 | 2.74  2.52 |  | 100  100 | 99.54  99.34 | 0.28  0.24 |  | 100  100 | 99.75  99.60 | 0.04  0.03 |
| 8 | 93  91 | 98.36  97.56 | 3.59  2.59 |  | 100  100 | 99.07  98.82 | 0.34  0.27 |  | 100  100 | 99.36  99.02 | 0.03  0.03 |
| 9 | 87  84 | 98.54  98.41 | 2.08  1.90 |  | 100  100 | 99.29  98.88 | 0.33  0.28 |  | 100  100 | 99.27  98.91 | 0.03  0.02 |
| 10 | 87  80 | 97.99  96.84 | 2.42  1.74 |  | 100  100 | 98.53  98.04 | 0.19  0.15 |  | 100  100 | 98.58  97.94 | 0.03  0.03 |
| 11 | 83  72 | 97.53  97.21 | 2.18  2.08 |  | 100  100 | 98.50  97.72 | 0.29  0.22 |  | 100  100 | 98.58  97.95 | 0.02  0.02 |
| 12 | 77  66 | 96.46  96.65 | 1.86  2.00 |  | 100  100 | 97.80  96.86 | 0.22  0.18 |  | 100  100 | 97.66  96.79 | 0.04  0.03 |
| 13 | 66  65 | 99.20  97.44 | 2.64  1.88 |  | 100  100 | 97.45  96.38 | 0.33  0.26 |  | 100  100 | 97.61  96.70 | 0.02  0.02 |
| 14 | 70  58 | 96.99  95.87 | 2.01  1.41 |  | 100  99 | 96.44  95.36 | 0.21  0.17 |  | 100  100 | 96.68  95.55 | 0.03  0.02 |
| 15 | 60  53 | 94.19  95.91 | 2.21  2.09 |  | 100  98 | 96.60  95.54 | 0.32  0.24 |  | 100  100 | 96.67  95.49 | 0.02  0.02 |
| 16 | 72  56 | 94.06  93.73 | 3.21  2.71 |  | 100  97 | 95.60  93.83 | 0.26  0.23 |  | 100  100 | 95.52  94.15 | 0.02  0.02 |
