# Supplementary material for: ConPADE: Genome Assembly Ploidy Estimation from Next-Generation Sequencing Data
Source: PLoS Comput Biol. 2015 Apr 16;11(4):e1004229. doi: 10.1371/journal.pcbi.1004229 (PMC4400156; doi:10.1371/journal.pcbi.1004229)
Supplement: S6 Table — Annotation results of 30 wheat (Triticum aestivum) contigs from chromosome arm 5D with an estimated ploidy of four. Results based on BLASTN alignments against the nucleotide database of NCBI (NT). (DOCX) [file pcbi.1004229.s013.docx]

**S6 Table:** Wheat annotation results. Putative annotation results of 30 wheat (*Triticum aestivum*) contigs from chromosome arm 5D with an estimated ploidy of four. Results based on BLASTN alignments against the nucleotide database of NCBI (NT).

| Contig | Subject GI | Subject Description | E value |
| --- | --- | --- | --- |
| 2179122 | 44888773 | *Aegilops tauschii* transposon Stowaway MITE | 1.1e-57 |
| 2347563 | 45357051 | *Triticum monococcum* transposon retroelement Artem | 2.8e-37 |
| 2896680 | 344222520 | *Secale cereale* ITS1 – 5.8S rRNA – ITS2 – 26S rRNA | 0.0 |
| 3279865 | 40841719 | *Oryza sativa* TCP-1/cpn60 chaperonin family protein | 0.0 |
| 4029532 | 51556884 | *T. aestivum* similar to Wilma LTR and Barbara LTR | 1.1e-69 |
| 4469718 | 44888773 | *A. tauschii* transposon Angela | 6.9e-13 |
| 4472075 | 63147801 | *Hordeum vulgare* transposon MITE, stowaway, Stolos-1 | 6.1e-49 |
| 4488335 | 11037259 | *S. cereale* retrotransposon Bilby (centromeric region) | 0.0 |
| 4488633 | 45357051 | *T. monococcum* transposon retroelement Artem | 4.8e-64 |
| 4488778 | 225166551 | *Lophopyrum elongatum* retrotransposon Pivu-1 | 0.0 |
| 4488923 | 44888773 | *A. tauschii* transposon XJ3 | 2.0e-47 |
| 4489133 | 45357051 | *T. monococcum* Inverted repeat-4 | 6.2e-28 |
| 4490343 | 76261904 | *Saccharum* hybrid transposon hopscotch-like | 2.8e-96 |
| 4491722 | 169649045 | *T. aestivum* cultivar Chinese Yumai mitochondrion | 0.0 |
| 4492258 | 45357051 | *T. monococcum* Copia retrotransposon Angela | 0.0 |
| 4492373 | 383215294 | *T. aestivum* retrotransposon Gypsy TREP | 9.2e-71 |
| 4492895 | 45357051 | *T. monococcum* transposon retroelement Jorge | 3.3e-148 |
| 4492905 | 34582228 | *H. roshevitzii* BARE-1 LTR | 0.0 |
| 4493483 | 380446985 | *Saccharum* hybrid transposon | 0.0 |
| 4494338 | 14289180 | *T. monococcum* transposon Ty3/gypsy DNA, centromeric region | 0.0 |
| 4495356 | 383215294 | *T. aestivum* transposon CACTA TREP 3004_Boris | 0.0 |
| 4496119 | 45357051 | *T. monococcum* Copia retrotransposon Angela | 7.0e-23 |
| 4496709 | 15088543 | *T. monococcum* truncated Gypsy-like retrotransposon Greti | 0.0 |
| 4496732 | 15028431 | *T. monococcum* WIS-2-1A copia-like retrotransposon | 1.2e-115 |
| 4497289 | 45357051 | *T. monococcum* Copia retrotransposon Angela | 0.0 |
| 4497537 | 45357051 | *T. monococcum* Gypsy retrotransposon Wham | 0.0 |
| 4497831 | 193074366 | *T. aestivum* transposon CACTA | 3.8e-33 |
| 4498503 | 45239090 | *T. aestivum* transposon | 3.2e-180 |
| 4498737 | 45357051 | *T. monococcum* Gypsy retrotransposon Fatima | 0.0 |
| 4498819 | 205362427 | *T. durum* Ty1-copia-like Ttd1.3 RT-RNaseH pseudogene | 0.0 |
